# System-Wide Characterization of MoArf GTPase Family Proteins and Adaptor Protein MoGga1 Involved in the Development and Pathogenicity of Magnaporthe oryzae

**DOI:** 10.1128/mBio.02398-19

**Published:** 2019-10-15

**Authors:** Shengpei Zhang, Lina Yang, Lianwei Li, Kaili Zhong, Wenhao Wang, Muxing Liu, Ying Li, Xinyu Liu, Rui Yu, Jialiang He, Haifeng Zhang, Xiaobo Zheng, Ping Wang, Zhengguang Zhang

**Affiliations:** a Department of Plant Pathology, College of Plant Protection, Nanjing Agricultural University, Nanjing, China; b Key Laboratory of Integrated Management of Crop Diseases and Pests, Ministry of Education, Nanjing, China; c Department of Pediatrics, Louisiana State University Health Sciences Center, New Orleans, Louisiana, USA; d Department of Microbiology, Immunology & Parasitology, Louisiana State University Health Sciences Center, New Orleans, Louisiana, USA; University of Texas Health Science Center

**Keywords:** ADP ribosylation factor, pathogenicity, vesicle trafficking, Golgi, *Magnaporthe oryzae*

## Abstract

ADP ribosylation factor (Arf) small GTPase family members are involved in vesicle trafficking and organelle maintenance in organisms ranging from Saccharomyces cerevisiae to humans. A previous study identified Magnaporthe oryzae Arf6 (MoArf6) as one of the Arf proteins that regulates growth and conidiation in the rice blast fungus M. oryzae, but the remaining family proteins remain unknown. Here, we identified six additional Arf proteins, including MoArf1, MoArl1, MoArl3, MoArl8, MoCin4, and MoSar1, as well as their sole adaptor protein, MoGga1, and determined their shared and specific functions. We showed that the majority of these proteins exhibit positive regulatory functions, most notably, in growth. Importantly, MoArl1, MoCin4, and MoGga1 are involved in pathogenicity through the regulation of host penetration and invasive hyphal growth. MoArl1 and MoCin4 also regulate normal vesicle trafficking, and MoCin4 further controls the formation of the biotrophic interfacial complex (BIC). Moreover, we showed that Golgi-cytoplasm cycling of MoArl1 is required for its function. Finally, we demonstrated that interactions between MoArf1 and MoArl1 with MoGga1 are important for Golgi localization and pathogenicity. Collectively, our findings revealed the shared and specific functions of Arf family members in M. oryzae and shed light on how these proteins function through conserved mechanisms to govern growth, transport, and virulence of the blast fungus.

## Abstract (precis)

Magnaporthe oryzae is the causal agent of rice blast, representing the most devastating diseases of rice worldwide, which results in losses of amounts of rice that could feed more than 60 million people each year. Arf (ADP ribosylation factor) small GTPase family proteins are involved in vesicle trafficking and organelle maintenance in eukaryotic cells. To investigate the function of Arf family proteins in M. oryzae, we systematically characterized all seven Arf proteins and found that they have shared and specific functions in governing the growth, development, and pathogenicity of the blast fungus. We have also identified the pathogenicity-related protein MoGga1 as the common adaptor of MoArf1 and MoArl1. Our findings are important because they provide the first comprehensive characterization of the Arf GTPase family proteins and their adaptor protein MoGga1 functioning in a plant-pathogenic fungus, which could help to reveal new fungicide targets to control this devastating disease.

## INTRODUCTION

Magnaporthe oryzae is the causal agent of rice blast. Understanding the pathogenesis of M. oryzae is therefore essential for disease management. In M. oryzae, cellular growth, development, and pathogenicity are regulated by G protein-mediated signal transduction pathways that govern a diverse array of processes, ranging from surface recognition to gene expression, cytoskeleton organization, and vesicle trafficking (1–5). In addition to the major heterotrimeric G proteins, eukaryotic cells also contain five families of monomeric small G proteins, including Ras, Rho, Rab, Ran, and Arf (6). Arf was first identified as a cofactor required for the ADP ribosylation of heterotrimeric G protein G_(s)_ by cholera toxin (7–9). The large Arf family includes three subfamilies: Arf proteins, Arf-like (Arl) proteins, and Sar proteins (10). The Arl proteins share high level of sequence conservation with Arf proteins, and the Sar proteins are also classified into the Arf family due to their N-terminal amphipathic helix and functional similarity to Arf proteins (11, 12). Typically of small G proteins, Arf proteins are cycled between the active GTP-bound and inactive GDP-bound forms through the functions of GEFs (guanine nucleotide exchange factors) and GAPs (GTPase-activating proteins) (12, 13). Arf proteins have an amphipathic helix and a myristoylated glycine site at the N terminus (11, 14), and they also possess a special interswitch to enable communication between the nucleotide-binding site and the N-terminal membrane-facing site (15).

On the basis of sequence homology, the Arf subfamily proteins are further divided into three classes. Class I is highly conserved among all eukaryotes, whereas class II occurs in metazoans, and class III occurs in metazoans and fungi (10, 12, 16). The Arl subfamily proteins have more members and functions than Arf proteins, and some are conserved among yeast, plants, and metazoans, while others occur only in vertebrates; members of the Sar protein subfamily are conserved among all eukaryotes (11, 12). We used Arf proteins to represent all of the Arf large families in our description. Similarly to the budding yeast Saccharomyces cerevisiae and human-pathogenic fungus Candida albicans, M. oryzae contains seven Arf proteins, including a human Arl2 homolog named MoCin4 (for “M. oryzae Cin4”) (6, 11, 17). Previous studies have established the roles of Arf proteins in regulating vesicle trafficking, organelle structures, phospholipid metabolism, and secretion and endocytosis in various systems (12, 18, 19). Recent studies in S. cerevisiae have also identified roles of S. cerevisiae Arl1 (ScArl1) and ScArl3 in the transport of the autophagy protein ScAtg9 (20, 21).

The Arf proteins activate vesicle transport by recruiting coat protein complex COPI, COPII, and most clathrin coat proteins (22). Due to the clathrin coats not directly binding to lipids, adaptors are needed for anchoring the budding membrane. There are four clathrin adaptor classes: AP-1 and AP-3, heterotetrameric APs, epsin-like proteins, and Gga (Golgi-localized, gamma-adaptin ear homology, Arf-binding) proteins (23, 24). The Arf interacts with Gga proteins such as yeast ScGga1 and ScGga2 and Homo sapiens Gga1 (HsGga1), HsGga2, and HsGga3 (25, 26). Gga proteins stabilize Arf1 in the GTP-bound form by inhibiting the GTPase activity of ArfGAP (27). In S. cerevisiae, ScArf1 also genetically interacts with ScDnm1 dynamin to regulate lipid transfer and mitochondrion morphology (28). Previous studies in filamentous fungi indicated that extension and invasion of hyphal tip growth require the long-distance transport of the membrane and proteins to the hyphal axis; for example, the conditional inactivation of ArfA in Aspergillus niger or SarA in A. nidulans impacts abnormal transport and hyphal morphology (29–32). Previous studies also showed that CaArf2 and CaArl1 are important in hyphal growth and virulence of C. albicans (17) and that MoArf6 and AnArfB are homologues of S. cerevisiae ScArf3 in M. oryzae and A. nidulans, respectively (33–35). Regardless, detailed studies of Arf proteins and their functional partner Gga proteins in phytopathogenic fungi remain lacking.

Previously, we demonstrated that ArfGAP protein MoGlo3 is involved in vesicle trafficking and pathogenicity in M. oryzae (36). We also previously characterized dynamin GTPase superfamily proteins and showed that MoDnm1 mediates peroxisomal and mitochondrial fission regulating vesicle trafficking and pathogenicity of the blast fungus (37). Here, we characterized all seven Arf proteins and demonstrated their important functions in the growth, development, and pathogenicity of the blast fungus. We also characterized MoGga1 as an Arf-interacting Gga protein important not only in Arf functions but also in conidiation and pathogenicity.

## RESULTS

### Identification of Arf proteins and MoGga1 from M. oryzae.

**FIG 1:**
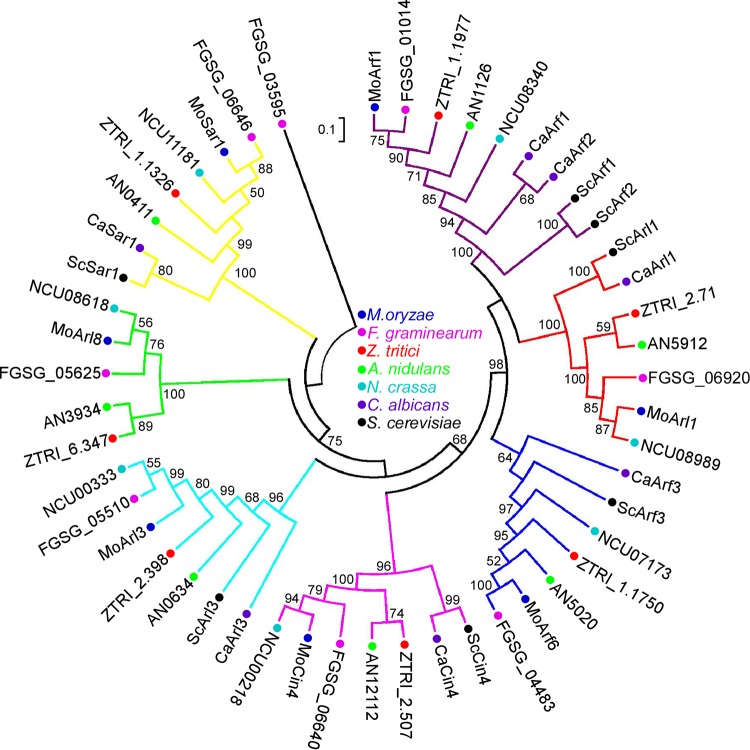
Phylogenetic analysis of putative Arf proteins in fungi. The Arf proteins from different fungi were aligned using the Clustal_W program, and the neighbor-joining tree was constructed with 1,000 bootstrap replicates by the use of MEGA 5.05. The sequences were obtained in the following organisms: M. oryzae, F. graminearum, Z. tritici, A. nidulans, N. crassa, C. albicans, and S. cerevisiae.

We searched for putative *ARF* genes in the available genome of M. oryzae (http://fungidb.org/fungidb/) and identified seven genes (MGG_04438, MGG_01574, MGG_08859, MGG_04976, MGG_10676, MGG_06362, and MGG_12887) that potentially encode Arf proteins. We first confirmed the expression of these genes by reverse transcription-PCR (RT-PCR), with the exception of MGG_12887, which was manually annotated to encode a protein of 181 amino acid residues (GenBank accession no. MG601752) (see Fig. S1A and B in the supplemental material). According to the protein sequences, a phylogenetic tree of the putative Arf proteins that included M. oryzae (7 proteins), Fusarium graminearum (8 proteins), Zymoseptoria tritici (7 proteins), A. nidulans (7 proteins), Neurospora crassa (7 proteins), C. albicans (7 proteins), and S. cerevisiae (7 proteins) was constructed. On the basis of the bootstrap values (of more than 50%), these proteins were classified into 8 clades, with the same clade grouped into the same colored branch (Fig. 1). The amino acid sequence alignment showed that all seven MoArf proteins contained the five conserved GTP/GDP-binding motifs (G1 to G5) as follows: in G1, GXXXXGK(S/T); in G2, XTX; in G3, DXXG; in G4, (N/T)(K/Q)D; in G5, (T/G/C)(C/S)A. MoArf1, MoArf6, and MoArl1 also have a conserved N-myristoylation motif (Fig. S1C).

10.1128/mBio.02398-19.1 FIG S1 Correction and sequence alignment of the MoArf proteins in M. oryzae. (A) Reverse transcription-PCR (RT-PCR) analysis of the putative *MoARF* genes. PCR products were obtained from the cDNA of the wild-type strain for MGG_04438, MGG_01574, MGG_08859, MGG_04976, MGG_10676, and MGG_06362 but not for MGG_12887, as the asterisk indicates. (B) The sequence of the predicted MGG_12887 gene model was suggested to be 618 bp; the sequence of the corrected gene model was 546 bp. F1/R1 and F2/R2 were the two primer pairs used to verify the gene model. The PCR products from wild-type cDNA could not be obtained using F1/R1 but could be obtained with F2/R2. M, marker. (C) Sequence alignment of the MoArf proteins in M. oryzae. The five conserved GTP/GDP motifs are indicated in G1, G2, G3, G4, and G5, and the conserved N-myristoylated motif is also indicated. Download FIG S1, TIF file, 1.8 MB. Copyright © 2019 Zhang et al. 2019 Zhang et al. This content is distributed under the terms of the Creative Commons Attribution 4.0 International license.

We also introduced MoArf1, MoArl1, MoArl3, and MoCin4 into the S. cerevisiae Δ*Scarf1*, Δ*Scarl1*, Δ*Scarl3*, and Δ*Sccin4* mutants, respectively, and found that they restored the sensitivity to various concentrations of hygromycin B (HygB) (Fig. S2). Although functional complementation of MoArf6, MoArl8, and MoSar1 was not possible, we nevertheless named them on the basis of the high sequence conservation and on data from previous studies (35, 38). Additionally, we identified MoGga1 (MGG_00852) as the sole Gga protein homolog of M. oryzae.

10.1128/mBio.02398-19.2 FIG S2 Functional complementation of S. cerevisiae Δ*Scarf* mutants. Serial dilutions of BY4741, Δ*Scarf* mutants, and Δ*Scarf* mutants transformed with pYES2 or pYES2-*MoARF* genes were grown on SD-Met-Leu-His-Ura (galactose) or SD-Met-Leu-His-Ura (galactose plus HygB) plates at 30°C for 4 days and then photographed. Download FIG S2, TIF file, 2.7 MB. Copyright © 2019 Zhang et al. 2019 Zhang et al. This content is distributed under the terms of the Creative Commons Attribution 4.0 International license.

### Expression patterns and targeted deletions of *MoARF* genes.

To characterize the functions of MoArf proteins, we performed qRT-PCR analysis to examine their transcriptional patterns in various growth stages. The transcript level of *MoARF1* was 3.8-fold higher at the conidial stage than that at the mycelium stage. The *MoARL1* transcription was upregulated during the early infection stage, with its transcript level increased by 20-, 55-, and 32-fold at 8, 24, and 48 h postinoculation (hpi), respectively, relative to that of the mycelium stage. The level of *MoSAR1* transcription was at least 18-fold higher than that seen at the mycelium stage in all observed infection stages. The transcriptional profiles of *MoARF6*, *MoARL3*, *MoARL8*, and *MoCIN4* remained relatively constant (Fig. S3A). These results indicated the relative importance of MoArf1 in conidiation and of MoArl1 and MoSar1 in infection.

10.1128/mBio.02398-19.3 FIG S3 Transcriptional patterns and target deletions of the related genes. (A) The phase-specific expression of *MoARF* genes was analyzed by quantitative real-time PCR (qRT-PCR) normalized to *ACTIN* (MGG_03982) with cDNA from vegetative hyphae, conidia, and infectious hyphae. Error bars show standard deviations of results from three replicates. (B) qRT-PCR analysis of the expression levels of *MoARF1* and *MoSAR1* under NaNO_3_-induced conditions. The relative expression levels of *MoARF1* and *MoSAR1* under NaNO_3_-induced conditions normalized to *ACTIN* (MGG_03982) were calculated. Asterisks indicate significant differences. (C) qRT-PCR analysis of the expression levels of *MoARF1* and *MoSAR1* under NaGlu. The relative expression levels of *MoARF1* and *MoSAR1* under conditions of repression in the presence of different concentrations of NaGlu normalized to *ACTIN* (MGG_03982) were calculated. Asterisks indicate significant differences. (D) qRT-PCR analysis of the expression levels of *MoARF1* and *MoSAR1* under conditions of NaGlu exposure in planta. The relative expression levels of *MoARF1* and *MoSAR1* under conditions of exposure to 460 mM NaGlu in planta for 3 and 5 days were calculated. (E) Schematic illustration and Southern blot analysis of targeted gene deletion. Arrows indicate the orientations of the targeted genes and the *HPH* (hygromycin phosphotransferase) genes. (F) Strategy and Southern blot analysis for the construction of CPR transformants. Promoter replacement cassettes were constructed by linking the flanking sequences of the target promoter with *HPH* and the promoter of *MoNIA1*. EI, EcoRI; EV, EcoRV; HIII, HindIII. Download FIG S3, TIF file, 1.4 MB. Copyright © 2019 Zhang et al. 2019 Zhang et al. This content is distributed under the terms of the Creative Commons Attribution 4.0 International license.

We have next obtained Δ*Moarf6*, Δ*Moarl1*, Δ*Moarl3*, Δ*Moarl8*, Δ*Mocin4*, and Δ*Mogga1* mutant strains by targeted gene disruption and verified their genotypes by Southern blotting (Fig. S3E). We also complemented the mutant strains with the respective wild-type (WT) genes that rescued all of the mutant defects. For reasons unknown, the Δ*Moarf1* and Δ*Mosar1* mutants could not be obtained despite the screening of ∼5,000 transformants each. Disruption of *ScARF1*/*ScARF2* and *ScSAR1* in S. cerevisiae, as well as *AnARFA* and *AnSAR1*, the respective paralogue of *ARF* and *SAR* in A. nidulans, was lethal (31, 39–41). To circumvent this, we introduced the nitrate reductase *MoNIA1* promoter, a conditional promoter described previously in studies of Z. tritici and Aspergillus fumigatus (42, 43), into *MoARF1* and *MoSAR1*. We obtained one Δ*Moarf1*/*CPR* mutant and one Δ*Mosar1*/*CPR* mutant, confirmed by Southern blotting (Fig. S3F), and found that the respective WT genes rescued the mutant defect.

The nitrate reductase promoter was induced by nitrate or other secondary nitrogen sources and repressed by ammonium or other primary nitrogen sources (43). We found that the levels of expression of *MoARF1* in the Δ*Moarf1*/*CPR* mutant and *MoSAR1* in the Δ*Mosar1*/*CPR* mutant were 4.9-fold and 15.1-fold compared to that in strain Guy11, respectively, under NaNO_3_ induction conditions (Fig. S3B). We next used NaGlu (C_5_H_8_NNaO_4_) as the sole nitrogen source and found that the level of expression of *MoARF1* in the Δ*Moarf1*/*CPR* mutant was about 0.5-fold lower than that in strain Guy11 at the concentrations tested (115, 230, and 460 mM). However, the expression levels of *MoSAR1* in the Δ*Mosar1*/*CPR* mutant were comparable to those in strain Guy11 at 115 mM and 230 mM NaGlu but not at 460 mM, where the expression level in the Δ*Mosar1*/*CPR* mutant was found to be 0.53-fold lower than that in Guy11 (Fig. S3C). For this reason, we selected 460 mM NaGlu for further studies.

### Characterization of growth and conidiation.

We examined vegetative growth on complete medium (CM), minimal medium (MM), oatmeal medium (OM), and straw decoction and corn (SDC) medium. A significant growth reduction was observed in the Δ*Moarf6*, Δ*Moarl1*, Δ*Moarl3*, and Δ*Mocin4* mutant strains but not the Δ*Moarl8* and Δ*Mogga1* mutants (see Table S1 in the supplemental material). We then examined the vegetative growth of the Δ*Moarf1*/*CPR* or Δ*Mosar1*/*CPR* mutant on MM containing 460 mM NaGlu as the sole nitrogen source and found that the mutants showed significantly reduced colony diameters compared to Guy11 and complemented strain Δ*Moarf1*/*CPR-C* or Δ*Mosar1*/*CPR-C* (Table S1). We also found the Δ*Moarf6*, Δ*Mocin4*, and Δ*Mogga1* mutants showed 0.57-, 0.02-, and 0.55-fold reductions, respectively, in conidiation compared with the WT Guy11 strain (Table S1).

10.1128/mBio.02398-19.9 TABLE S1 Phenotypic analysis among strains. Download Table S1, DOCX file, 0.02 MB. Copyright © 2019 Zhang et al. 2019 Zhang et al. This content is distributed under the terms of the Creative Commons Attribution 4.0 International license.

### MoArl1, MoCin4, and MoGga1 are required for full virulence.

**FIG 2:**
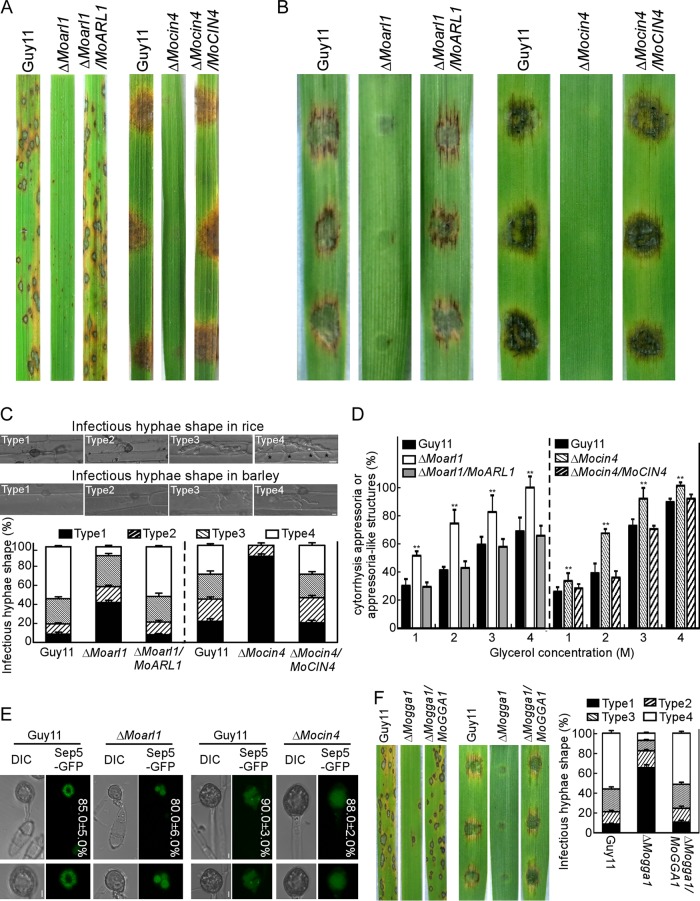
MoArl1, MoCin4, and MoGga1 are required for full virulence. (A) Pathogenicity assay in rice. Two-week-old rice seedlings were inoculated with related conidial suspensions (Δ*Moarl1*) or mycelia (Δ*Mocin4*) and photographed at 7 days postinoculation (dpi). (B) Pathogenicity assay in barley. One-week-old detached barley leaves were inoculated with the conidial suspension or mycelia and photographed at 5 dpi. (C) Penetration assay in rice cells at 48 hpi and in barley cells at 36 hpi. The appressorium (in rice) or appressorium-like (in barley) penetration sites (*n* = 100) were divided into types 1 to 4. Error bars represent standard deviations of results from three replicates. Black asterisks indicate hyphae extended to neighboring cells. Bar, 10 μm. (D) Statistical analysis of appressoria (Δ*Moarl1*) or appressorium-like structures (Δ*Mocin4*) revealed cytorrhysis in different glycerol concentrations. Asterisks represent significant differences. (E) The localization of Sep5-GFP in appressoria (Δ*Moarl1*) or appressorium-like structures (Δ*Mocin4*). Bar, 10 μm. DIC, differential inference contrast. (F) Pathogenicity and penetration assays for Δ*Mogga1* mutant. The criteria of the classification were the same as those described for Δ*Moarl1*. Error bars represent standard deviations of results from three replicates.

Spraying with the equal conidial suspensions, pin-sized specks were observed on leaves incubated with the Δ*Moarl1* mutant, in contrast to the WT blast lesions caused by the Δ*Moarf6*, Δ*Moarl3*, and Δ*Moarl8* mutants (Fig. 2A; see also Table S1). A similar result was observed in a detached barley leaf assay (Fig. 2B). As conidiation was severely impaired in the Δ*Mocin4* strain, hyphae were used for inoculation of detached rice and barley leaves, resulting in a nearly complete absence of disease symptoms (Fig. 2A and B; see also Table S1). Conidial suspensions of Δ*Moarf1*/*CPR* and Δ*Mosar1*/*CPR* mutants with 460 mM NaGlu were also tested for pathogenicity. The mutants caused typical disease symptoms similar to those caused by Guy11 and complemented strains in the injected-rice assays (Table S1). However, there were no differences between the Δ*Moarf1*/*CPR*, Δ*Mosar1*/*CPR*, and Guy11 strains in the expression levels of *MoARF1* or *MoSAR1* in planta (Fig. S3D). Nevertheless, our results suggested that MoArl1 and MoCin4 are important in pathogenicity.

To further examine MoArl1 and MoCin4 functions in infection, we carried out the penetration and colonization assay. In rice sheath infection, we observed 100 penetration sites and rated them from type 1 to type 4 (type 1, no penetration; type 2, penetrating peg formed; type 3, spreading but limited to one cell; type 4, spreading to neighboring cells) at 48 hpi. In the Guy11 and Δ*Moarl1*/*MoARL1* strains, more than 90% appressoria penetrated the rice cells, with more than 75% consisting of type 3 and type 4. In contrast, more than 50% of the penetration sites were type 1 and type 2 in the Δ*Moarl1* mutant (Fig. 2C). To determine the possible reasons for the reduced pathogenicity of the Δ*Mocin4* mutant, mycelia of the Guy11, Δ*Mocin4*, and Δ*Mocin4*/*MoCIN4* strains were inoculated onto detached barley leaves. We observed 100 penetration sites and also rated these from type 1 to type 4 (type 1, no penetration; type 2, penetrating peg formed; type 3, two or three invasive hyphae; type 4, more than three invasive hyphae) at 36 hpi. More than 45% of the penetration sites displayed type 3 and type 4 in the Guy11 and Δ*Mocin4*/*MoCIN4* strains, whereas more than 90% of the appressorium-like structures could not penetrate the barley cells in the Δ*Mocin4* mutant (Fig. 2C). These results demonstrated that MoArl1 and MoCin4 are involved in both penetration and colonization of the host cells.

As appressorium-mediated host penetration requires strong turgor pressure, we examined the turgor of appressorium or appressorium-like structures using a cytorrhysis assay (44). The results showed that the Δ*Moarl1* and Δ*Mocin4* mutants displayed a higher collapse rate than strain Guy11 and the complemented strains, indicating that MoArl1 is required for the normal turgor of appressorium and MoCin4 for that of appressorium-like structures (Fig. 2D). The septin (Sep) ring is required for turgor generation in M. oryzae (45), we therefore expressed Sep5-GFP (Sep5-green fluorescent protein) in Δ*Moarl1* and Δ*Mocin4* mutants. The appressorium showed a Sep5-GFP ring in Guy11 but a disorganized mass in the Δ*Moarl1* mutant; however, the appressorium-like structures did not exhibit a Sep5-GFP ring in either Guy11 or the Δ*Mocin4* mutant (Fig. 2E). Additionally, we also found that the Δ*Mogga1* mutant showed reduced pathogenicity compared with Guy11 and the complemented strain (Fig. 2F).

### MoArl1 and MoCin4 are required for normal vesicle trafficking.

**FIG 3:**
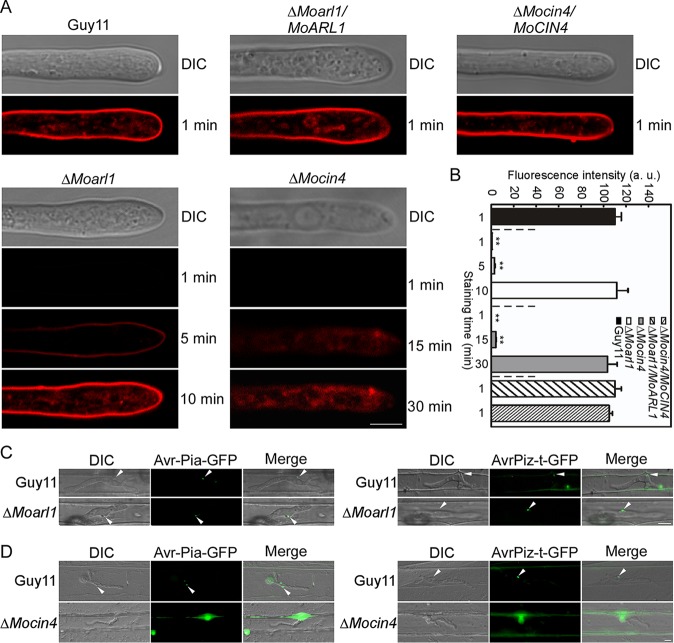
MoArl1 and MoCin4 are required for normal vesicle trafficking. (A) Hyphae of the strains were stained with FM4-64 for different minutes. Bar, 5 μm. (B) The integrated fluorescent density was calculated with ImageJ. Asterisks indicate significant differences compared with Guy11. a.u., arbitrary units. (C and D) Images of BICs and the BIC-accumulating cytoplasmic effector Avr-Pia-GFP and AvrPiz-t-GFP in rice (Δ*Moarl1*) (C) and barley (Δ*Mocin4*) (C) cells. Arrows indicate BICs. Bar, 10 μm.

To investigate the roles of MoArl1 and MoCin4 in vesicle trafficking, we examined the uptake of endocytic tracer FM4-64 dye. FM4-64 was internalized by Guy11 and the complemented strains after 1 min of incubation, but no definitive dye staining pattern was observed in the Δ*Moarl1* and Δ*Mocin4* mutants. After 5 min for the Δ*Moarl1* mutant and 15 min for the Δ*Mocin4* mutant, uptake of the stains was seen, and until 10 min for the Δ*Moarl1* mutant and 30 min for the Δ*Mocin4* mutant, the level of staining was comparable to that seen with Guy11 (Fig. 3A and B). This result suggested that MoArl1 and MoCin4 are required for endocytic uptake of FM4-64.

A recent study showed that vesicle trafficking is required for maintaining the plant-fungus interface and effector secretion (46); therefore, we investigated the roles of MoArl1 and MoCin4 in those processes. Guy11, Δ*Moarl1*, and Δ*Mocin4* strains were transformed with GFP-labeled Avr-Pia and AvrPiz-t, which preferentially accumulated in a biotrophic interfacial complex (BIC) and translocated to the plant cell cytoplasm (47). We found that both Avr-Pia and AvrPiz-t accumulated in BIC in the rice cells infected by the Guy11 and Δ*Moarl1* strains (Fig. 3C). In barley cells, we also observed that Avr-Pia and AvrPiz-t accumulated in BIC in Guy11 but that more than 80% ± 5.0% of Δ*Mocin4*-infected cells showed no observable BIC and Avr-Pia and AvrPiz-t were not detected (Fig. 3D). These results indicated that MoCin4 is required for BIC formation and for normal effector deployment.

### MoCin4 is involved in the scavenging of reactive oxygen species (ROS).

**FIG 4:**
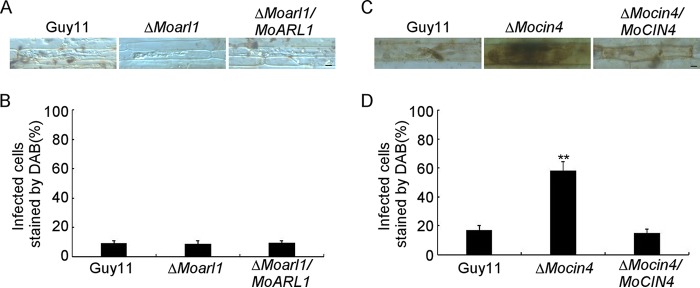
MoCin4 is involved in the scavenging of reactive oxygen species. (A and B) DAB was used to stain the sheaths injected with related conidial suspensions for Δ*Moarl1* cells and the stained cells were statistically analyzed. (C and D) DAB was used to stain the barleys infected with related mycelia for Δ*Mocin4* cells and statistically analyzed. Asterisks indicate a significant difference. Bar, 10 μm.

Plants protect themselves against pathogens by evolving ROS, while pathogens evolve effector and antioxidation systems to neutralize ROS (48–51). Since MoArl1 and MoCin4 function in vesicle trafficking and are required for pathogenicity, we measured the level of host ROS production using 3,3′-diaminobenzidine (DAB). We found that 12% of the rice cells infected by the Δ*Moarl1* mutant stained brown, similarly to Guy11 and the complemented strain (Fig. 4A and B). However, 58% of barley cells infected by the Δ*Mocin4* mutant stained brown compared to 18% and 17% of those infected by the Guy11 strain and the complemented strain, respectively (Fig. 4C and D).

### MoArl1 is localized to the cytoplasm and the Golgi, whereas MoCin4 is restricted to the cytoplasm.

**FIG 5:**
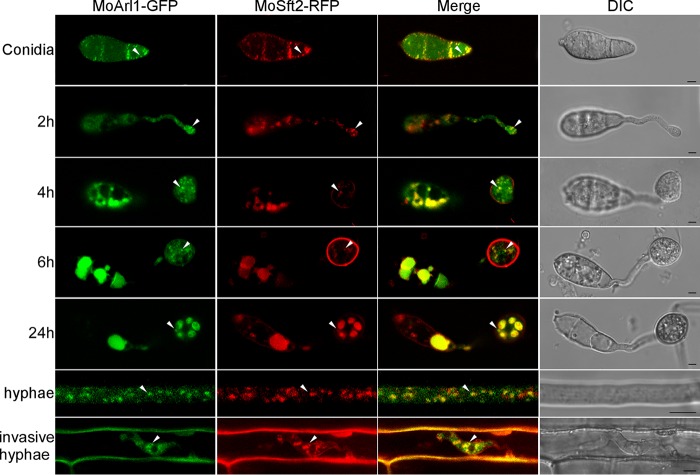
MoArl1 is localized to the Golgi and the cytoplasm. MoArl1 partially colocalizes with MoSft2 in conidia, germ tubes, appressoria, and vegetative and invasive hyphae. MoSft2 was expressed as a Golgi marker, and images were observed with confocal fluorescence microscopy (Zeiss LSM710 laser scanning microscope; 63× oil). Arrowheads show the representative colocalized areas. Bar, 5 μm.

To detect the localizations of MoArl1 and MoCin4, the native promoter of *MoARL1* and the entire *MoARL1* gene were fused with the green fluorescent protein (GFP). For MoCin4, since we could not detect any GFP (data not shown) using its native promoter, we opted to use the strong constitutively activated ribosomal protein 27 (RP27) promoter (52). MoArl1 was distributed throughout the cytoplasm but it also appeared as green fluorescence punctate. Since S. cerevisiae Arl1 is Golgi localized (53, 54), we tested whether the punctate colocalizes with the Golgi. To do this, we introduced the Golgi marker protein MoSft2-RFP (MoSft2-red fluorescent protein) (36, 55) and found that the green fluorescence punctate indeed colocalized with MoSft2-RFP in conidia, germ tubes, appressoria, and the vegetative and invasive hyphae (Fig. 5). To quantify the efficiency of colocalization, the images were subjected to Pearson’s colocalization analysis, which yielded the values of 0.39 ± 0.02 and 0.38 ± 0.01, respectively, in conidia and hyphae. MoCin4 appeared to be distributed throughout the cytoplasm (Fig. S4). The expression levels of MoArl1 and MoCin4 were verified by Western blotting (Fig. S5A and B).

10.1128/mBio.02398-19.4 FIG S4 MoCin4 distributed through all of the cytoplasms. The localization of different alleles for MoCin4 was observed using confocal fluorescence microscopy (Zeiss LSM710 laser scanning microscope; 63× oil). Bar, 5 μm. Download FIG S4, TIF file, 0.3 MB. Copyright © 2019 Zhang et al. 2019 Zhang et al. This content is distributed under the terms of the Creative Commons Attribution 4.0 International license.

10.1128/mBio.02398-19.5 FIG S5 Expression of related MoArl1, MoCin4, and MoGga1 alleles. (A and B) Western blot analysis of the point-mutated MoArl1 and MoCin4 alleles, introduced in Guy11, with the anti-GFP antibody. (C) Expression of the related MoGga1 protein. Western blotting was used for analysis of the level of expression of the corresponding MoGga1 protein with the anti-GFP antibody. Arrows indicate the protein sizes, and the size of the free GFP is also marked. Download FIG S5, TIF file, 0.5 MB. Copyright © 2019 Zhang et al. 2019 Zhang et al. This content is distributed under the terms of the Creative Commons Attribution 4.0 International license.

### Localization of MoArl1 is nucleotide dependent.

**FIG 6:**
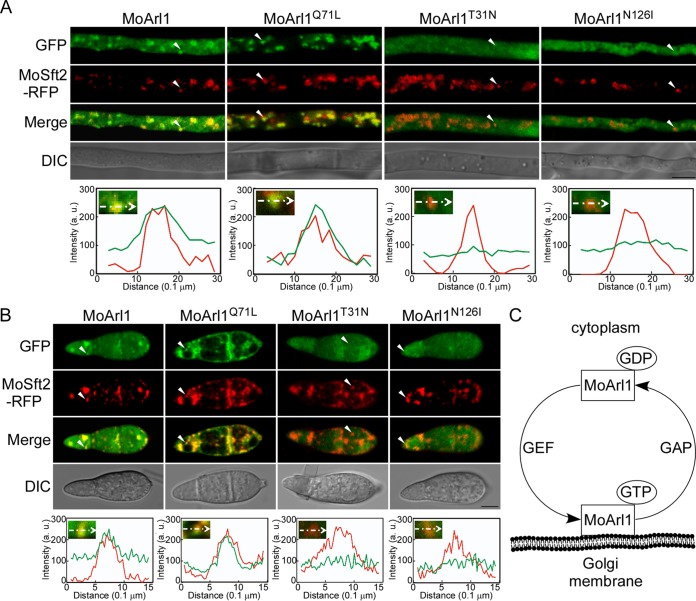
The localization of MoArl1 is nucleotide dependent. (A and B) Localization patterns of different forms of MoArl1 in hyphae (A) and conidia (B). Arrowheads show the areas used for determinations of fluorescence intensity profiles by line-scan analysis. Green lines stand for the fluorescence intensity of related MoArl1-GFP results and red for MoSft2-RFP. (C) Model of the association/disassociation of MoArl1 with the Golgi membrane. Bar, 5 μm.

To further study the conserved GTP/GDP binding motifs for localization of MoArl1, we observed the localization pattern of three point-mutated alleles, namely, MoArl1^T31N^-GFP, MoArl1^Q71L^-GFP, and MoArl1^N126I^-GFP, with results that showed dominant-negative, constitutively active, and allele-altering nucleotide exchange rates of MoArl1, respectively (53, 56). We found that MoArl1^Q71L^-GFP colocalized with MoSft2-RFP with weak cytosolic distributions in hyphae and conidia and that the Pearson’s colocalization values were 0.71 ± 0.06 and 0.69 ± 0.04, compared with 0.38 ± 0.02 and 0.40 ± 0.03 for MoArl1 and MoSft2, respectively (Fig. 6A and B). In contrast, we found that MoArl1^T31N^-GFP and MoArl1^N126I^-GFP lost the punctate signal distribution pattern and that the Pearson’s values were 0.03 ± 0.01/0.05 ± 0.02 and 0.08 ± 0.03/0.04 ± 0.02, respectively (Fig. 6A and B). The results reported above implied a recycled model for MoArl1 in which the GTP-bound form is associated with the Golgi and disassociates from the Golgi upon hydrolyzation into the GDP-bound form. The latter reassociates with the Golgi while being activated to the GTP-bound form (Fig. 6C).

Also, we observed localizations of three corresponding alleles for MoCin4, namely, MoCin4^T28N^-GFP, MoCin4^Q68L^-GFP, and MoCin4^N123I^-GFP. We found that they were all distributed throughout the cytoplasm (Fig. S4). The point-mutated alleles for MoArl1 and MoCin4 were transformed into Guy11, and their expression levels were confirmed by Western blot analysis (Fig. S5A and B).

### The GTP/GDP binding motifs are important for functions of MoArl1 and MoCin4.

**FIG 7:**
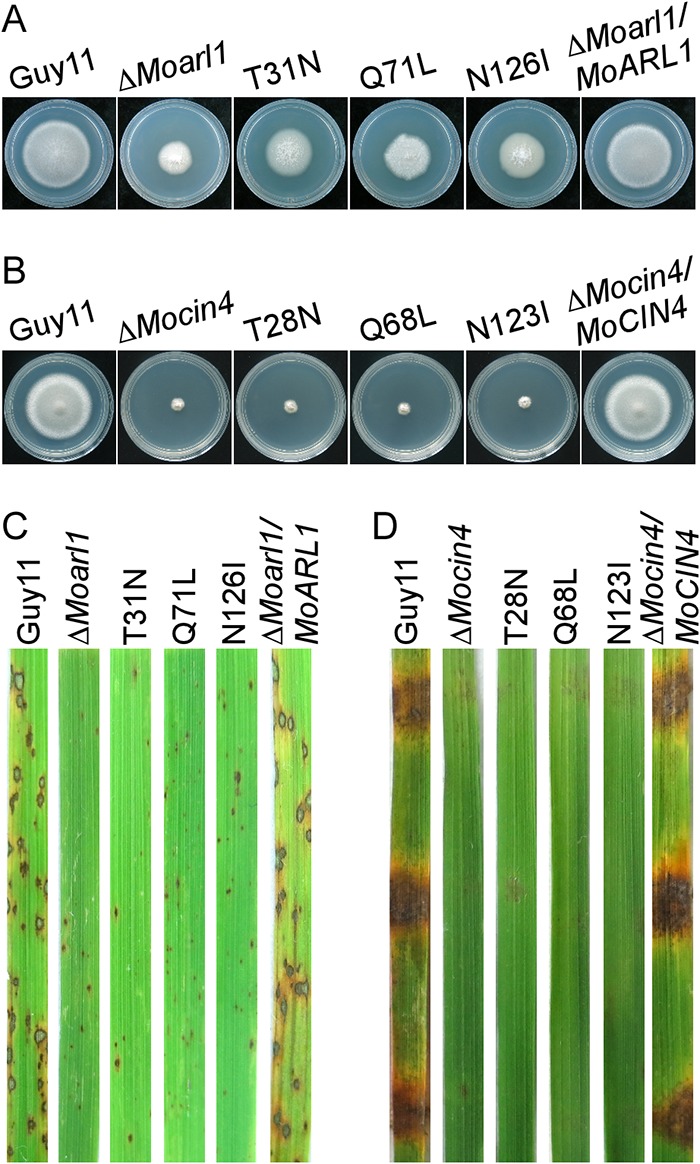
The GTP/GDP binding motifs are important for the functions of MoArl1 and MoCin4. (A) Colony morphology of Δ*Moarl1*-related strains after 7 days of incubation with CM plates. (B) Colony morphology of Δ*Mocin4*-related strains after 7 days of incubation with CM plates. (C) Rice spraying assay of the Δ*Moarl1*-related strains. (D) Detached rice leaf assay of the Δ*Mocin4*-related strains.

To investigate the function of the GTP/GDP binding motifs of MoArl1 and MoCin4, we created six point-mutated strains, namely, MoArl1^T31N^, MoArl1^Q71L^, MoArl1^N126I^, MoCin4^T28N^, MoCin4^Q68L^, and MoCin4^N123I^, which were obtained by the transformation of the point-mutated alleles into the Δ*Moarl1* or Δ*Mocin4* mutant. The differences between the WT and Δ*Moarl1* strains in the growth rates of MoArl1^T31N^, MoArl1^Q71L^, and MoArl1^N126I^ were moderate (Fig. 7A; see also Fig. S6A), and the growth rates of MoCin4^T28N^, MoCin4^Q68L^, and MoCin4^N123I^ were similar to those seen with the Δ*Mocin4* mutant (Fig. 7B; see also Fig. S6C). In the sprayed-rice assay, the MoArl1^T31N^, MoArl1^Q71L^, and MoArl1^N126I^ mutated strains showed pin-sized specks similar to those produced by the Δ*Moarl1* strain (Fig. 7C). In the detached rice leaf assay, MoCin4^T28N^, MoCin4^Q68L^, and MoCin4^N123I^ produced by the Δ*Mocin4* mutant caused barely any disease symptoms (Fig. 7D). The expression levels of the MoArl1^T31N^, MoArl1^Q71L^, MoArl1^N126I^, and ΔMoarl1/MoARL1 alleles were analyzed by Western blotting (Fig. S6D). For the MoCin4^T28N^, MoCin4^Q68L^, MoCin4^N123I^, and Δ*Mocin4*/*MoCIN4* strains, whose expression could not be monitored by Western blotting, we employed RT-PCR (Fig. S6E). The results suggested that GTP/GDP binding motifs are important for MoArl1 and MoCin4 functions.

10.1128/mBio.02398-19.6 FIG S6 Expression and growth tests of motif mutation strains. (A and B) Statistical analysis of colony diameters for the Δ*Moarl1*-related strains following 7 days of incubation in CM plates. (C) Statistical analysis of colony diameters for the Δ*Mocin4*-related strains. (D) Western blot analysis of MoArl1 expression in motif-specific mutation strains with the anti-GFP antibody. (E) RT-PCR analysis of transcription of *MoCIN4* in motif-specific mutation strains. Download FIG S6, TIF file, 0.3 MB. Copyright © 2019 Zhang et al. 2019 Zhang et al. This content is distributed under the terms of the Creative Commons Attribution 4.0 International license.

### The N-myristoylation motif is essential for function and Golgi localization of MoArl1.

**FIG 8:**
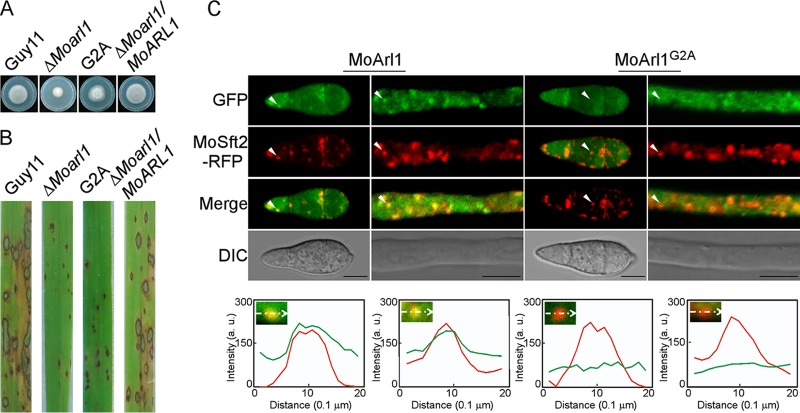
The N-myristoylated motif is essential for functions and Golgi localization of MoArl1. (A) Colony morphology of Δ*Moarl1*-related strains after 7 days of incubation with CM plates. (B) Rice spraying assay of the Δ*Moarl1*-related strains. (C) Localization pattern of different forms of MoArl1 in conidia and hyphae. Arrowheads show the areas used for determinations of fluorescence intensity profiles by line-scan analysis. Green lines stand for the fluorescence intensity of related MoArl1-GFP results and red for MoSft2-RFP. Bar, 5 μm.

In addition to the GTP/GDP binding motif, MoArl1 also has an N-myristoylation motif. We mutated the conserved glycine to alanine (MoArl1^G2A^) at the myristoyl acceptor site. The MoArl1^G2A^ mutant exhibited a moderate level of growth between those seen with the WT and Δ*Moarl1* strains (Fig. 8A; see also Fig. S6B) and showed pin-sized specks, similar to those seen with the Δ*Moarl1* mutant, in the sprayed-rice assay (Fig. 8B). We also found that MoArl1^G2A^-GFP had lost the colocalization pattern with MoSft2-RFP both in conidia and hyphae, and the Pearson’s values were 0.05 ± 0.01 and 0.06 ± 0.02 compared with 0.38 ± 0.05 and 0.36 ± 0.02 for MoArl1 and MoSft2, respectively (Fig. 8C). The level of expression of the MoArl1^G2A^ allele was verified by Western blotting (Fig. S5A and S6D). These results suggested an essential role of the N-myristoylation motif in the Golgi localization and function of MoArl1.

### MoGga1 interacts with both MoArl1 and MoArf1 in the Golgi.

**FIG 9:**
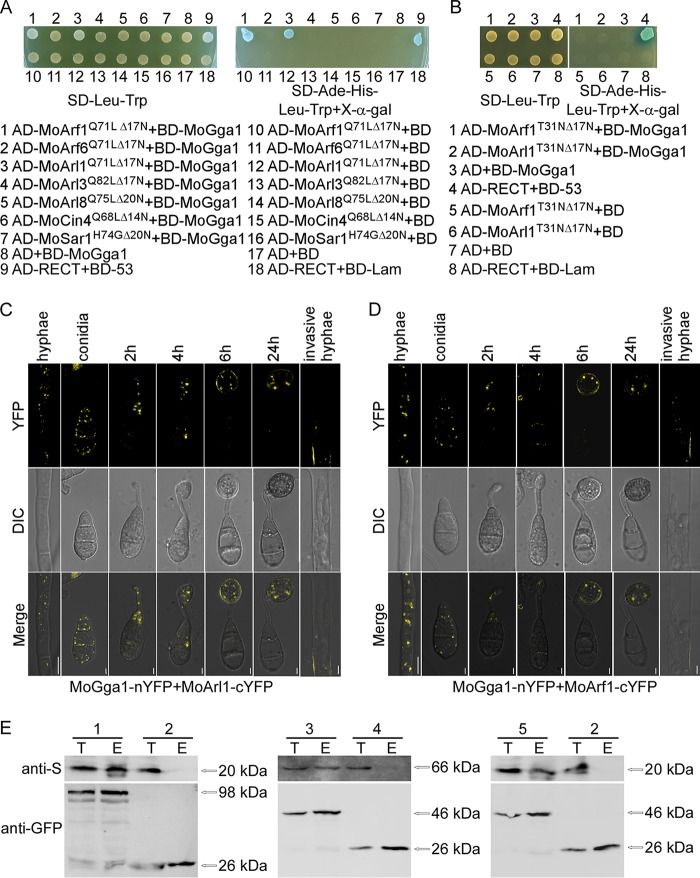
MoGga1 interacts with both MoArl1 and MoArf1. (A and B) Y2H assay for the interaction between the constitutively active (A) and dominant negative (B) forms of MoArf proteins with MoGga1. The yeast transformants expressing the bait and prey constructs were incubated on SD-Leu-Trp plates. The β-galactosidase activity was assayed on SD-Ade-His-Leu-Trp plates with X-Gal (5-bromo-4-chloro-3-indolyl-β-d-galactopyranoside). (C and D) BiFC assays for the interaction of MoArl1 (C) or MoArf1 (D) with MoGga1 in vivo. The transformants coexpressing MoGga1-YFP^N^ and MoArl1-YFP^C^ or MoArf1-YFP^C^ were observed in different developmental stages with confocal fluorescence microscopy (Zeiss LSM710 laser scanning microscope; 63× oil). Bar, 5 μm. (E) Co-IP assays for the interactions of MoArl1, MoGga1, and MoArf1. The coexpressing proteins (lanes 1, MoArl1-S/MoGga1-GFP; lanes 2, MoArl1-S/GFP; lanes 3, MoGga1-S/MoArf1-GFP; lanes 4, MoGga1-S/GFP; lanes 5, MoArl1-S/MoArf1-GFP) were extracted individually as the total proteins (T). Total proteins were eluted from the anti-GFP beads (E) and analyzed by Western blotting with anti-S and anti-GFP antibodies.

The ScArl1 yeast regulates three pathways in the Golgi, including the transport of ScGas1 to the plasma membrane, the targeting of ScImh1 with the Golgi, and the recruitment of ScGga to the Golgi (57, 58). Among them, Gga cooperates with ScArl1^Q71L^ to favor its interactions with downstream proteins during vesicle transport (26). We sought to investigate whether such functional relationships exist in M. oryzae by performing the yeast two-hybrid (Y2H) assay. Since previous studies showed that full-length *ARF* sequences contain membrane binding domains that may interfere with Y2H (53, 59, 60), a truncated form, lacking the first 17 N-terminal hydrophobic amino acids, was used instead. Y2H revealed an interaction between MoArl1^Q71LΔ17N^ and MoGga1. In addition, we tested the interactions of the other six truncated GTP-bound MoArf proteins with MoGga1 and showed that MoArf1^Q71LΔ17N^, but not truncated GDP-bound MoArl1^T31NΔ17N^ and MoArf1^T31NΔ17N^, also interacted with MoGga1 (Fig. 9A and B).

To examine whether the interactions also exist in vivo, we employed the bimolecular fluorescence complementation (BiFC) assay. The transformants coexpressing MoGga1-YFP^N^ (N-terminal MoGga1-yellow fluorescent protein) and MoArl1-YFP^C^ exhibited punctate yellow signals in conidia, germ tubes, appressoria, and vegetative and invasive hyphae (Fig. 9C). The similar punctate signals were also observed in the transformants coexpressing MoGga1-YFP^N^ and MoArf1-YFP^C^ (Fig. 9D). The interactions between MoGga1 and MoArl1 and between MoGga1 and MoArf1 were further validated by the in vivo coimmunoprecipitation (Co-IP) assay, and the results indicated that both MoArl1 and MoArf1 interact with MoGga1 and that MoArl1 also interacts with MoArf1 in vivo (Fig. 9E). We also tested the interactions among all 7 Arf proteins; however, we did not find interactions within the Arf family members (Fig. S7), with the exception of the interaction between MoArl1 and MoArf1 described above.

10.1128/mBio.02398-19.7 FIG S7 Co-IP assays for the interactions within the MoArf family. The coexpressing proteins were extracted individually as the total proteins (T). Total proteins were eluted from the anti-GFP beads (E) and analyzed by Western blotting with anti-S and anti-GFP antibodies. The protein pairs were analyzed as follows: panel 1, MoArl1-S/MoSar1-GFP; panel 2, MoArf6-S/MoSar1-GFP; panel 3, MoArf1-S/MoSar1-GFP; panel 4, MoCin4-S/MoSar1-GFP; panel 5, MoArl1-S/MoArf6-GFP; panel 6, MoArf1-S/MoArf6-GFP; panel 7, MoCin4-S/MoArf6-GFP; panel 8, MoCin4-S/MoArl1-GFP; panel 9, MoCin4-S/MoArf1-GFP; panel 10, MoArf1-S/MoArl8-GFP; panel 11, MoSar1-S/MoArl8-GFP; panel 12, MoArf6-S/MoArl8-GFP; panel 13, MoCin4-S/MoArl8-GFP; panel 14, MoArl3-S/MoArl8-GFP; panel 15, MoArl1-S/MoArl8-GFP; panel 16, MoCin4-S/MoArl3-GFP; panel 17, MoSar1-S/MoArl3-GFP; panel 18, MoArf6-S/MoArl3-GFP; panel 19, MoArl1-S/MoArl3-GFP; panel 20, MoArf1-S/MoArl3-GFP. Download FIG S7, TIF file, 1.0 MB. Copyright © 2019 Zhang et al. 2019 Zhang et al. This content is distributed under the terms of the Creative Commons Attribution 4.0 International license.

We further observed the localization of MoGga1 and found that it also appears as green punctate, in a manner similar to that seen with the GTP-bound MoArl1, and that most of the GFP signals colocalized with MoSft2-RFP. The Pearson’s values were 0.48 ± 0.06 and 0.45 ± 0.03 in conidia and hyphae, respectively (Fig. S8A). Additionally, we observed that MoArf1 partially localized to the Golgi in conidia and hyphae and that the Pearson’s values were 0.35 ± 0.02 and 0.36 ± 0.03, respectively (Fig. S8B). The results reported above led us to investigate whether the yellow punctate fluorescence seen with the interaction (Fig. 9) represents Golgi structures. We observed their colocalization with MoSft2-RFP and found that they colocalized with each other, with Pearson’s values of 0.42 ± 0.05 and 0.41 ± 0.03 for the strains coexpressing MoGga1-YFP^N^, MoArl1-YFP^C^, and MoSft2-RFP and 0.43 ± 0.04 and 0.44 ± 0.02 for the strains coexpressing MoGga1-YFP^N^, MoArf1-YFP^C^, and MoSft2-RFP strains in conidia and hyphae, respectively (Fig. S8C and D). These results suggested that MoGga1 interacts with MoArl1 and MoArf1 in the Golgi.

10.1128/mBio.02398-19.8 FIG S8 MoGga1 interacts with MoArl1 and MoArf1 in the Golgi. (A) MoGga1 is localized to the Golgi. MoGga1 colocalizes with MoSft2 in conidia and hyphae. (B) MoArf1 is localized to the Golgi and the cytoplasm. MoArf1 partially colocalizes with MoSft2 in conidia and hyphae. (C) The yellow punctate signals of strains coexpressing MoGga1-nYFP and MoArl1-cYFP were colocalized with MoSft2-RFP. (D) The yellow punctate signals of strains coexpressing MoGga1-nYFP and MoArf1-cYFP were colocalized with MoSft2-RFP. Images were observed using confocal fluorescence microscopy (Zeiss LSM710 laser scanning microscope; 63× oil). Arrows show the representative colocalized areas. Bar, 5 μm. Download FIG S8, TIF file, 1.2 MB. Copyright © 2019 Zhang et al. 2019 Zhang et al. This content is distributed under the terms of the Creative Commons Attribution 4.0 International license.

### The localization and function of MoGga1 are dependent on its interaction with MoArf1 and MoArl1.

**FIG 10:**
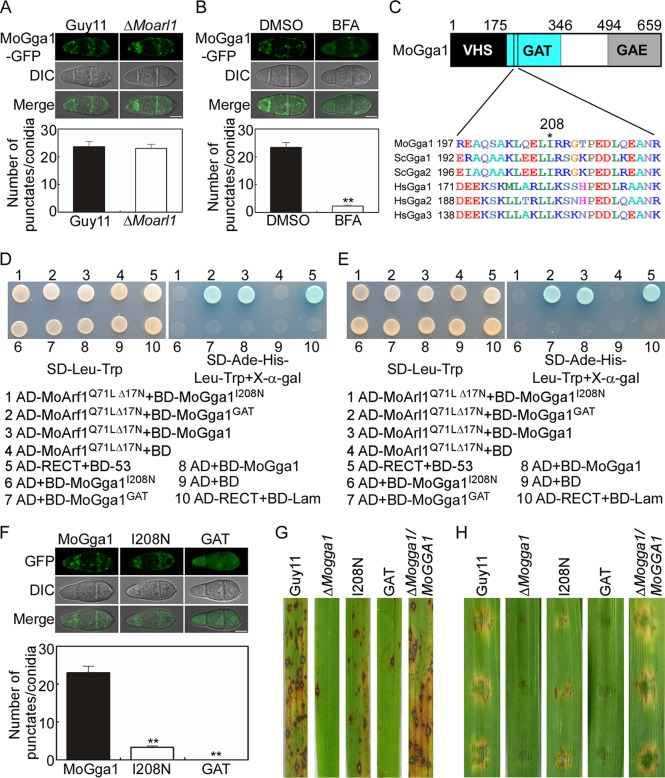
Localization and function of MoGga1 are dependent on its interaction with MoArf1 and MoArl1. (A) The localization of MoGga1-GFP in the Guy11 and Δ*Moarl1* mutant strains. The averaged GFP punctate numbers determined for 50 conidia were counted and analyzed. Images were observed with confocal fluorescence microscopy (Zeiss LSM710 laser scanning microscope; 63× oil). Bar, 5 μm. (B) Localization of MoGga1-GFP in Guy11 following BFA treatment. The averaged GFP punctate numbers determined for 50 conidia were counted and analyzed. Asterisks indicate significant differences. Images were observed with confocal fluorescence microscopy (Zeiss LSM710 laser scanning microscope; 63× oil). Bar, 5 μm. (C) Structure and domain prediction of MoGga1. Regions of the domains are indicated by amino acid numbers. The asterisk indicates the conserved leucine or isoleucine residue of MoGga1 relative to that in ScGgas and HsGgas. (D and E) Y2H assay for interactions between the point mutation MoGga1^I208N^ or GAT domain with the constitutively active forms of MoArf1 (D) and MoArl1 (E). (F) Localization of point-mutated MoGga1^I208N^-GFP and truncated MoGga1^GAT^-GFP in conidia. The averaged GFP punctate numbers determined for 50 conidia were counted and analyzed. Asterisks indicate significant differences. Images were observed using confocal fluorescence microscopy (Zeiss LSM710 laser scanning microscope; 63× oil). Bar, 5 μm. (G) Rice spraying assays for the Δ*Mogga1*-related mutants. (H) Detached barley assays for the Δ*Mogga1*-related mutants.

To examine how MoGga1 is recruited to the Golgi, we first observed the localization of MoGga1 in the Δ*Moarl1* mutant and found no significant differences in punctate signals between the mutant and Guy11 (Fig. 10A). Since a Δ*Moarf1* mutant was not available, we treated the WT Guy11 strain containing MoGga1-GFP with brefeldin A (BFA), which inhibits the exchange of GDP to GTP for Arf proteins (61). The punctate GFP signals were significantly reduced following treatment (Fig. 10B), suggesting that localization of MoGga1 is dependent on MoArf proteins.

We found that MoGga1 contains conserved VHS, GAT, and GAE domains (Fig. 10C). The GAT domain was previously identified as an Arf-binding domain, and point mutations in the GAT domain were shown to affect its interaction with Arf proteins (62–64). We identified isoleucine in MoGga1 at position 208 (Fig. 10C), which corresponds to the critical residue present in other Gga proteins (64). We then changed the isoleucine 208 residue to asparagine and found that MoGga1^I208N^ failed to interact with MoArf1^Q71L^ and MoArl1^Q71L^ (Fig. 10D and E). Meanwhile, we also found that MoGga1^GAT^ (GAT domain of MoGga1 alone) still interacted with MoArf1^Q71L^ and MoArl1^Q71L^ (Fig. 10D and E).

To test the functions of these interactions, we fused MoGga1^I208N^ and MoGga1^GAT^ with a GFP tag before the transformation of the Δ*Mogga1* mutant. We obtained strains MoGga1^I208N^ and MoGga1^GAT^ (verified by Western blotting; Fig. S5C) and found that the MoGga1^I208N^ strain showed dramatically decreased levels of punctate GFP signals and that the MoGga1^GAT^ strain exhibited homogeneous GFP signals throughout the cytoplasm (Fig. 10F). The effect of point and domain mutations was further determined by infection tests. The MoGga1^I208N^ strain resulted in some lesions, which were less extensive than those seen with Guy11 but more extensive than those seen with the Δ*Mogga1* mutant. The MoGga1^GAT^ strain caused lesions that were similar in extent to those seen with the Δ*Mogga1* mutant (Fig. 10G and H). These results indicated that interactions between MoGga1 and MoArf1/MoArl1 are required but not sufficient for the localization and the function of MoGga1.

## DISCUSSION

The Arf GTPase family proteins are involved in many cellular processes, including vesicle trafficking, cytoskeletal organization, signaling transduction, and organelle maintenance in diverse organisms ranging from yeast to animals (11, 28). However, the functions of Arf GTPases in filamentous plant pathogens remain poorly understood. We found that Arf proteins of M. oryzae have specific as well as shared functions governing the growth, development, and pathogenicity of the fungus. Specifically, MoArf6 and MoCin4 are involved in growth and conidiation. MoArl1 and MoArl3 are positive regulators of vegetative growth, whereas MoArl8 is dispensable for most of the functions tested. Additionally, experiments that used a strategy employing a conditional promoter led to the conclusion that MoArf1 and MoSar1 are also important for growth.

We previously found that ArfGAP protein MoGlo3, mediating endocytosis and vesicle trafficking, regulates growth, conidiation, and pathogenesis in the blast fungus (36). Intriguingly, interactions between MoGlo3 and all MoArf proteins cannot be established by the Y2H assay (data not shown). Because M. oryzae is a fungal pathogen, we focused our research efforts on pathogenicity and found that MoArl1 and MoCin4 are required for the full virulence of the fungus. The Δ*Moarl1* and Δ*Mocin4* mutants showed reduced virulence due to the defect in appressorial penetration and invasive hyphal growth. But the mechanisms responsible for the reduced pathogenicity of the two mutants also showed some differences; Δ*Moarl1* mutants exhibited lower appressorial turgor levels and disorganized septin rings, while Δ*Mocin4* mutants exhibited lower appressorial turgor levels and defects in scavenging of ROS. A recent study in C. albicans revealed a novel role of CaArl1 in virulence (17), and our characterization of MoArl1 is in accordance with that finding. In addition, since MoCin4 is homologous to yeast ScCin4 and human HsArl2, which regulate normal microtubule stability (65) and mitochondrial and microtubule morphology (66, 67), respectively, our identification of MoCin4 from M. oryzae may represent the first study result indicating that modulation of such a protein can also impact virulence.

MoArl1 is localized to the Golgi and the cytoplasm, consistent with previously performed studies of other systems (53, 62). Our results also revealed that MoArl1^Q71L^-GFP localizes to the Golgi more efficiently than MoArl1-GFP does and that MoArl1^T31N^-GFP and MoArl1^N126I^-GFP are cytosolic. The MoArl1^Q71L^ form is likely to be locked in the constitutively activated GTP-bound state, whereas the MoArl1^T31N^ and MoArl1^N126I^ forms likely maintain the GDP-bound state (53, 56). We demonstrated that the localization of MoArl1 in the Golgi and the cytoplasm is nucleotide dependent and could be regulated by GEFs and GAPs (12, 13). Moreover, we also showed that MoArl1^G2A^-GFP is distributed throughout the cytoplasm. Together with the incomplete recovery of these point-mutated isoforms for suppressing the defect in growth and pathogenicity of the Δ*Moarl1* mutant, we propose that the cycled localization of MoArl1 between the Golgi and the cytoplasm is essential for its normal function.

The Golgi functions as the central hub in the conventional secretory pathway of fungi that sorts protein cargos to the plasma membrane, extracellular cells, or the recycled system (30, 68, 69). To further elucidate the functions of MoArl1 with respect to the Golgi, we identified the sole Gga protein, MoGga1, as a MoArl1^Q71L^-interacting protein in M. oryzae. There are some contradictions between our results and those obtained with this interaction in other systems; one study showed that GTP-bound ScArl1 could not interact with ScGga1 or ScGga2 (63), whereas other studies revealed that ScArl1 and ScGga2 may interact with each other, either directly or indirectly, and that ScGga2 functions as a monomeric adaptor protein of ScArl1 in clathrin coat formation (26, 70). Our results demonstrated that MoArl1^Q71L^, but not MoArl1^T31N^, directly interacts with MoGga1. On the basis of the cytoplasm localization of MoArl1^T31N^, the Golgi localization of MoArl1^Q71L^, and interaction of MoArl1^Q71L^ with MoGga1 on the Golgi, we concluded that MoArl1 is localized to the Golgi, where it interacts with MoGga1 for function.

The localization of ScGga2 was previously shown to exhibit a slight change in the Δ*Scarl1* mutant in S. cerevisiae (26). Our results showed that MoArl1 does not affect the localization of MoGga1. Again, previous studies have indicated a central role of human HsArf1 in the recruitment of HsGga1, HsGga2, and HsGga3 (25, 71). On the basis of the finding that both MoArl1^Q71L^ and MoArf1^Q71L^ interacted with MoGga1 and that the levels of MoGga1-GFP punctate signals were markedly reduced following treatment with BFA, we hypothesized that MoArl1 and MoArf1 cooperate to interact and recruit MoGga1 to the Golgi.

Human HsGga proteins must interact with HsArf proteins for proper localization and function, whereas these interactions play a minor role in the Golgi localization and in the function of ScGga proteins in S. cerevisiae (27, 64). This raises the issue of how this is different in M. oryzae. Point mutation of the common binding area for MoArf1 and MoArl1 in MoGga1 abrogated its interaction with both proteins and dramatically affected its localization, resulting in an incomplete recovery of the pathogenicity defect of the Δ*Mogga1* mutant. These results indicated the importance of the interactions between MoGga1 with MoArf1 and MoArl1 for the proper localization and function of MoGga1. Taking the data together, we have demonstrated that MoGga1, whose localization and function depend on its interaction with MoArf1 and MoArl1, acts as the common adaptor of MoArf1 and MoArl1. The growing identification of specific and common proteins interacting with Arf proteins indicated that Arf GTPase family members do not only work alone but also can cross talk with each other (53, 72, 73).

In summary, we have characterized seven Arf GTPase family members that regulate growth, development, and pathogenicity in M. oryzae and we have also identified MoGga1 as a sole common adaptor protein for MoArf1 and MoArl1. Further investigations of MoArf proteins and their additional interacting partners are warranted to elucidate the dynamic and multiple networks of this important group of small GTPase proteins in M. oryzae.

## MATERIALS AND METHODS

### Strains and culture conditions.

M. oryzae Guy11 was used as the wild-type strain in this study. All strains were cultured on CM agar plates in the dark at 28°C, unless indicated otherwise. The strains were incubated in liquid CM for 2 days in darkness for extraction of DNA, RNA, and protein.

### Phylogenetic tree construction and sequence alignment.

All of the Arf proteins of M. oryzae, F. graminearum, Z. tritici, A. nidulans, N. crassa, C. albicans, and S. cerevisiae were obtained from the NCBI database (https://www.ncbi.nlm.nih.gov/) or from FungiDB (http://fungidb.org/fungidb/). The phylogenetic tree was constructed using MEGA 5.05 programs with 1,000 bootstrap replicates and the neighbor-joining method. The alignment of MoArf proteins was performed by the use of CLUSTAL_W programs.

### Growth, conidiation, and turgor assays.

For vegetative growth, small blocks of strains were cultured on the plates of CM, OM, MM, and SDC for 7 days and then measured and analyzed (74). For the growth of the CPR mutant, the NaNO_3_ of MM was replaced by 460 mM NaGlu. For conidiation assay, the strains were cultured on SDC in the dark for 7 days, followed by 3 days of constant illumination under fluorescent light, and then the conidia were collected and analyzed (75, 76). The turgor assays for appressorium or appressorium-like structures were as described previously (77, 78).

### S. cerevisiae Δ*Scarf* mutant complementation.

The full-length cDNAs of MoArf proteins, which were amplified using primers (see Table S2 in the supplemental material), were cloned into pYES2 vector using the GAL1 promoter, induced by galactose treatment, and repressed by glucose treatment. After sequencing, the fused constructs were transformed into the corresponding S. cerevisiae Δ*Scarf* mutants (BY4741 mutants Δ*YDL192W*, Δ*YOR094W*, Δ*YBL164C*, Δ*YPL051W*, and Δ*YMR138W*). Putative transformants were selected on Sabouraud dextrose (SD) medium lacking uracil. For complementation assays, yeast strains were cultured in liquid yeast extract-peptone-dextrose (YPD) overnight and were diluted to an optical density at 600 nm (OD_600_) of 0.1, and then 5-μl volumes of 10-fold serial dilutions were grown on SD-Met-Leu-His-Ura (galactose) plates with or without HygB. The S. cerevisiae Δ*Scarf* mutants and BY4741-expressed pYES2 strains were controls.

10.1128/mBio.02398-19.10 TABLE S2 Primers used in this study. Download Table S2, DOC file, 0.03 MB. Copyright © 2019 Zhang et al. 2019 Zhang et al. This content is distributed under the terms of the Creative Commons Attribution 4.0 International license.

### Reverse transcription-PCR (RT-PCR), quantitative RT-PCR, and gene expression analysis.

For RT-PCR, RNA was reverse transcribed into first-strand cDNA with a reverse transcription kit (Vazyme). The correction of the putative *MoARF* gene model was performed with the primers (Table S2). The qRT-PCR was performed with an Applied Biosystems 7500 real-time PCR system as described previously (79). The relative quantification transcriptional levels of all *MoARF* genes were normalized to that of *ACTIN* (MGG_03982).

### Gene deletion, complementation, and amino acid substitution.

The gene deletion mutants were generated by the standard one-step gene replacement strategy as previously described (80). The CPR deletion mutants were generated as described previously in Z. tritici and A. fumigatus (42, 43). Two approximately 1.0-kb DNA fragments flanking the promoter of *MoARF1* or *MoSAR1* and the promoter of *MoNIA* were amplified using the primer pairs (Table S2). The downstream flanking sequence and the promoter of *MoNIA* were further amplified by overlap PCR. The upstream flanking sequence and overlapped PCR products were digested by restriction endonucleases and ligated with the same enzymes used to cleave pCX62 vector, respectively. The verified plasmids were transformed into Guy11, and putative mutants were screened by PCR and further confirmed by Southern blotting.

For complementation and amino acid substitution experiments, the complement fragments, which contained the related genes and their 1.5-kb native promoters, were amplified with primer pairs (Table S2) and cotransformed with XhoI-digested pYF11 vector (bleomycin resistance) into the yeast XK1-25 strain (52) and then transformed into the Escherichia coli DH5α strain for further amplification. After sequencing, the fused-pYF11 plasmids were transformed into the related mutant for the corresponding complementation (47).

### Pathogenicity assays.

Equal volumes of conidial suspensions (5 × 10^4^ conidia/ml) with 0.2% (wt/vol) gelatin were inoculated on rice seedlings (Oryza sativa cv. CO39) or detached barleys. Inoculated plants were kept in the dark under conditions of 90% humidity for the first 24 h and then subjected to light/dark cycles for 5 to 7 days (81). For analysis of the pathogenicity of mutants with fewer conidia, mycelia cultured in liquid CM for 2 to 4 days were washed and inoculated onto the detached barleys or rice leaves as described previously (78, 82). For analysis of the pathogenicity of CPR mutants, the conidial suspensions were supplemented with 460 mM NaGlu for the rice injection sheath assay (47, 83). For the microscopic observation of penetration and invasive hyphae in plant tissues, conidia or mycelia were infected with rice sheaths or barley leaves and plant cells were microscopically observed after 36 h (barley leaves) or 48 h (rice sheaths) of inoculation.

### Endocytosis and secretion assays.

For endocytosis, the strains were cultured in liquid CM for 24 h and then stained with FM4-64 for several minutes. For secretion, the conidia or mycelia were used to infect rice sheath or barleys and then BICs and the localization of Avr-Pia and AvrPiz-t in the infected cells were observed.

### DAB staining.

The infected rice sheaths or barley were incubated with 1 mg/ml DAB for 8 h, and the stained cells were subjected to washing in an ethanol/acetic acid solution (47:1 [vol/vol]) for 4 h and then observed.

### Subcellular localization observation.

To observe the subcellular localization, all of the proteins were fused on a GFP tag with their native promoters, with the exception of MoCin4, which showed no GFP signal; thus, the RP27 promoter was instead. All of the images were observed using confocal fluorescence microscopy (Zeiss LSM710 laser scanning microscope; 63× oil).

### Yeast two-hybrid assays.

The truncated point-mutated MoArf proteins were cloned into pGADT7 as the prey constructs and MoGga1 was cloned into pGBKT7 as the bait construct using primers (Table S2). After sequencing, the prey and bait constructs were transformed into yeast strain AH109 in pairs. The Trp-positive (Trp^+^) and Leu^+^ transformants were isolated and assayed for growth on SD-Trp-Leu-His-Ade medium and for expression of the LacZ reporter gene (84).

### Bimolecular fluorescence complementation (BiFC) assays.

The *MoGGA1*-YFP^N^ plasmid was constructed by cloning the *MoGGA1* gene with its native promoter into pHZ65 vector. Similarly, the *MoARL1*-YFP^C^ and *MoARF1*-YFP^C^ plasmids were constructed by cloning the corresponding gene into pHZ68 vector. Construct pairs (the *MoGGA1*-YFP^N^ and *MoARL1*-YFP^C^ pair and the *MoGGA1*-YFP^N^ and *MoARF1*-YFP^C^ pair) were transformed into the protoplasts of Guy11. The transformants were selected by the use of both hygromycin and bleomycin and were then observed by confocal fluorescence microscopy (Zeiss LSM710 laser scanning microscope; 63× oil).

### Coimmunoprecipitation (Co-IP) assay.

The *MoARF* and *MoGGA1* genes with their corresponding native promoters were cloned into both pXY203 (S tag) vector and pYF11 (GFP tag) vector with primers (Table S2). After sequencing, construct pairs were transformed into the protoplasts of Guy11. The total proteins were extracted from transformants coexpressing the above two fusion constructs and incubated with anti-GFP beads (Abmart). After three washes, the elution of the proteins bound to anti-GFP beads was analyzed by Western blotting with anti-GFP (Abmart) (1:5,000) and anti-S (Abcam) (1:5,000) antibodies (85).

### Brefeldin A (BFA) treatment.

The harvested conidia were treated with 5 μg/ml BFA (Sigma) dissolved in dimethyl sulfoxide (DMSO) for 2 min, and the DMSO treatment acted as a control. The treated conidia were then observed using confocal fluorescence microscopy (Zeiss LSM710 laser scanning microscope; 63× oil).

### Data availability.

The GenBank accession numbers (species names) for organisms used in this study are as follows: XP_003713533.1 (M. oryzae MoArf1); XP_003715902.1 (M. oryzae MoArf6); XP_003712475.1 (M. oryzae MoArl1); XP_003713882.1 (M. oryzae MoArl3); XP_003714552.1 (M. oryzae MoArl8); MG601752 (M. oryzae MoCin4); XP_003717215.1 (M. oryzae MoSar1); NP_010089.1 (S. cerevisiae ScArf1); NP_010144.1 (S. cerevisiae ScArf2); NP_014737.1 (S. cerevisiae ScArf3); NP_009723.3 (S. cerevisiae ScArl1); NP_015274.1 (S. cerevisiae ScArl3); NP_013858.1 (S. cerevisiae ScCin4); NP_015106.1 (S. cerevisiae ScSar1); XP_716284.1 (C. albicans CaArf1); XP_723175.1 (C. albicans CaArf2); XP_019330830.1 (C. albicans CaArf3); XP_722675.1 (C. albicans CaArl1); XP_713902.2 (C. albicans CaArl3); XP_721425.2 (C. albicans CaCin4); XP_019331008.1 (C. albicans CaSar1); XP_003718126.1 (M. oryzae MoGga1); NP_010645.1 (S. cerevisiae ScGga1); NP_011976.1 (S. cerevisiae ScGga2); NP_037497.1 (H. sapiens HsGga1); AAF05708.1 (H. sapiens HsGga2); NP_054720.1 (H. sapiens HsGga3). Gene sequences of the fungal strains used in this study are available at FungDB (http://fungidb.org/fungidb/) under the indicated accession numbers: F. graminearum FGSG_01014, F. graminearum FGSG_04483, F. graminearum FGSG_06920, F. graminearum FGSG_06640, F. graminearum FGSG_05625, F. graminearum FGSG_06646, F. graminearum FGSG_05510, F. graminearum FGSG_03595, N. crassa NCU08340, N. crassa NCU07173, N. crassa NCU089890, N. crassa NCU00218, N. crassa NCU08618, N. crassa NCU11181, N. crassa NCU00333, Z. tritici ZTRI_1.1977, Z. tritici ZTRI_1.1750, Z. tritici ZTRI_2.71, Z. tritici ZTRI_2.507, Z. tritici ZTRI_6.347, Z. tritici ZTRI_1.1326, Z. tritici ZTRI_2.398, N. crassa AN1126, N. crassa AN5020, N. crassa AN5912, N. crassa AN12112, N. crassa AN3934, N. crassa AN0411, and N. crassa AN0634.
