# Supplementary material for: System-Wide Characterization of MoArf GTPase Family Proteins and Adaptor Protein MoGga1 Involved in the Development and Pathogenicity of Magnaporthe oryzae
Source: mBio. 2019 Oct 15;10(5):e02398-19. doi: 10.1128/mBio.02398-19 (PMC6794486; doi:10.1128/mBio.02398-19)
Supplement: TABLE S1 [file mBio.02398-19-st001.docx]

**Table S1. Phenotypic analysis among strains**

| Strain | Colony diameter (cm)^a^ | | | | | Conidiation (×100/cm^2^)^c^ | Pathogenicity (Diseased area %) | | |
| --- | --- | --- | --- | --- | --- | --- | --- | --- | --- |
|  | CM | MM | OM | SDC | MM^b^ |  | d | e | f |
| Guy11 | 4.7±0.1^A^ | 4.1±0.1^A^ | 4.8±0.1^A^ | 4.0±0.1^A^ | 3.5±0.1^A^ | 121.8±1.5^A^ | 36.8±2.0^A^ | 34.8±2.5^A^ | 37.2±1.8^A^ |
| Δ*Moarf6* | 3.0±0.1^C^ | 2.7±0.1^B^ | 3.0±0.1^C^ | 3.0±0.1^B^ | ND | 69.4±1.8^B^ | 38.4±2.5 ^A^ | ND | ND |
| Δ*Moarl1* | 2.1±0.2^D^ | 2.0±0.2^C^ | 2.3±0.1^D^ | 2.5±0.1^B^ | ND | 120.4±1.6^A^ | 0.8±0.1^C^ | ND | ND |
| Δ*Moarl3* | 3.5±0.1^B^ | 2.4±0.1^B^ | 3.7±0.3^B^ | 2.8±0.5^B^ | ND | 119.9±1.8^A^ | 39.9±2.8 ^A^ | ND | ND |
| Δ*Moarl8* | 4.7±0.1^A^ | 3.8±0.3^A^ | 4.7±0.1^A^ | 4.3±0.2^A^ | ND | 121.6±2.2^A^ | 35.4±3.0^A^ | ND | ND |
| Δ*Mocin4* | 1.0±0.1^E^ | 0.8±0.1^D^ | 0.7±0.1^E^ | 0.6±0.1^C^ | ND | 2.4±0.2^C^ | ND | 4.1±0.5 ^B^ | ND |
| Δ*Mogga1* | 4.6±0.1^A^ | 4.1±0.3^A^ | 4.8±0.2^A^ | 3.9±0.3^A^ | ND | 66.8±0.3^B^ | 1.7±0.2 ^B^ | ND | ND |
| Δ*Moarf1/CPR* | ND | ND | ND | ND | 1.5±0.1^B^ | ND | ND | ND | 37.3±2.3^A^ |
| Δ*Mosar1/CPR* | ND | ND | ND | ND | 1.5±0.1^B^ | ND | ND | ND | 35.0±1.6^A^ |
| Δ*Moarf1/CPR-C* | ND | ND | ND | ND | 3.4±0.1^A^ | ND | ND | ND | 36.5±2.6^A^ |
| Δ*Mosar1/CPR-C* | ND | ND | ND | ND | 3.4±0.1^A^ | ND | ND | ND | 38.2±3.0^A^ |

^a^Colony diameter was measured after the strains cultured on CM, MM, OM and SDC medium for 7 days.

^b^Colony diameter was measured after the strains cultured on MM with the NaNO_3_ was substituted by NaGlu for 7 days.

^c^Conidiation was analyzed after the strains cultured on SDC medium for 10 days.

^d^Diseased leaf area was analyzed after rice seedlings were inoculated with conidial suspensions for 7 days. Data show the percentage of lesion areas analyzed by ImageJ.

^e^Diseased leaf area was analyzed by ImageJ after rice seedlings were inoculated with mycelia for 7 days.

^f^Diseased leaf area was analyzed by ImageJ after conidial suspensions with 460mM NaGlu were injected into rice sheaths for 7 days.

Mean and standard deviations (±SD) was calculated from three replicates, different letters indicate statistically significant differences (*p* < 0.01). ND = not determined.
