# Supplementary figures and images for: System-Wide Characterization of MoArf GTPase Family Proteins and Adaptor Protein MoGga1 Involved in the Development and Pathogenicity of Magnaporthe oryzae
Source: mBio. 2019 Oct 15;10(5):e02398-19. doi: 10.1128/mBio.02398-19 (PMC6794486; doi:10.1128/mBio.02398-19)

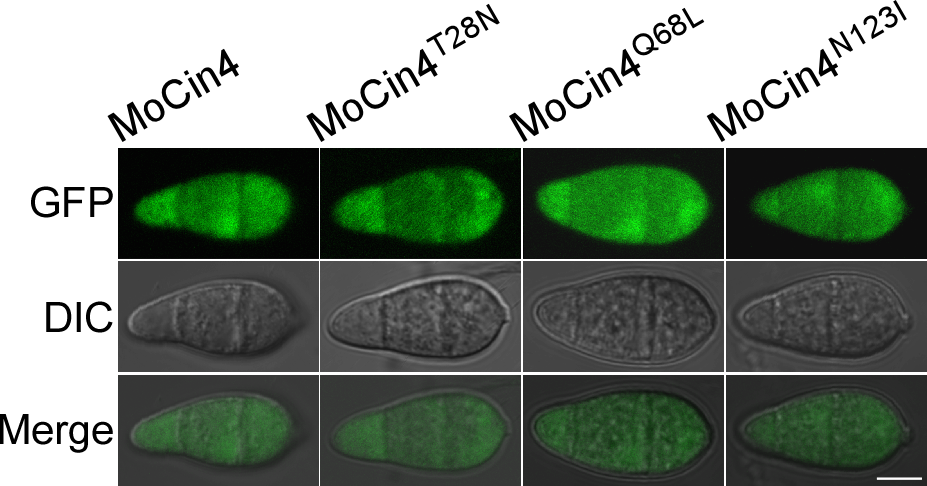

Supplement: FIG S4 [file mBio.02398-19-sf004.tif]

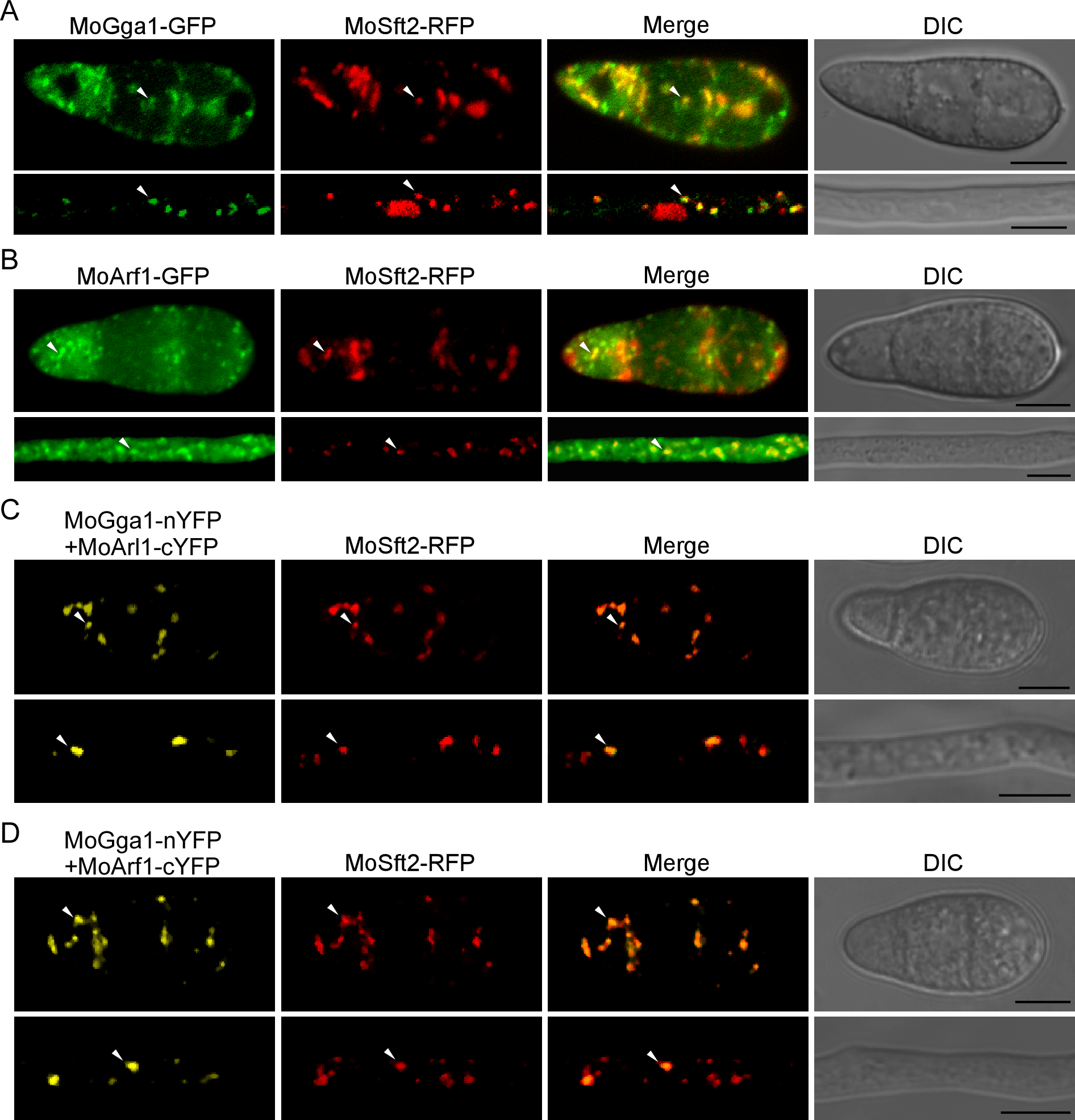

Supplement: FIG S8 [file mBio.02398-19-sf008.tif]
