# Supplementary material for: System-Wide Characterization of MoArf GTPase Family Proteins and Adaptor Protein MoGga1 Involved in the Development and Pathogenicity of Magnaporthe oryzae
Source: mBio. 2019 Oct 15;10(5):e02398-19. doi: 10.1128/mBio.02398-19 (PMC6794486; doi:10.1128/mBio.02398-19)
Supplement: TABLE S2 [file mBio.02398-19-st002.doc]

**Table S2. Primers used in this study**

| **Primer name** | | **Sequence (5’-3’)** | **Purpose** |
| --- | --- | --- | --- |
| MGG_12887F1 | | ATGATCGTACGGAAACGCAAAGAGA | Correct MGG_12887 ORF |
| MGG_12887R1 | | TCACCCTTCGTAATCGATCGTCTTG | Correct MGG_12887 ORF |
| MGG_12887F2 | | ATGTTGTCAATCCTCAGGAAAGC | Correct MGG_12887 ORF |
| MGG_12887R2 | | CTAATATAGGAACAACCTAGC | Correct MGG_12887 ORF |
| MGG_04438F | | ATGGGTCTCAGCATCAGCAAG | Correct MGG_04438 ORF |
| MGG_04438R | | TTACTGGTGGCCTGCCTTGC | Correct MGG_04438 ORF |
| MGG_01574F | | ATGGCTGCCATCATCAAAG | Correct MGG_01574 ORF |
| MGG_01574R | | TCATTTGCTAGAGTACTTCATCAAC | Correct MGG_01574 ORF |
| MGG_08859F | | ATGTATCACCTAGCTAAAGGACTGT | Correct MGG_08859 ORF |
| MGG_08859R | | TCACCTCATTACAGGCGGTCTGCTC | Correct MGG_08859 ORF |
| MGG_04976F | | ATGGGACAAAGTGTTTCATGGCT | Correct MGG_04976 ORF |
| MGG_04976R | | TTATGACTCTTGCGAGACCGTTTGC | Correct MGG_04976 ORF |
| MGG_10676F | | ATGGGAGGGCAGCTCT | Correct MGG_10676 ORF |
| MGG_10676R  MGG_06362F  MGG_06362R | | TCACTTCTTGGCCGGCGCAG  ATGTGGATCATCAACTGGTTTTACG  CTACACATACTGCGAAAGCCATCTG | Correct MGG_10676 ORF  Correct MGG_06362 ORF  Correct MGG_06362 ORF |
| *MoARF1*pYES2F | | CGGGATCCATGGGTCTCAGCATCAGCAAG | amplify *MoARF1* sequence |
| *MoARF1*pYES2R | | GCTCTAGATTACTGGTGGCCTGCCTTGC | amplify *MoARF1* sequence |
| *MoARF6*pYES2F | | CGGAATTCATGGGAGGGCAGCTCTCCAAGATGA | amplify *MoARF6* sequence |
| *MoARF6*pYES2R  *MoARL1*pYES2F  *MoARL1*pYES2R  *MoARL3*pYES2F  *MoARL3*pYES2R  *MoCIN4*pYES2F  *MoCIN4*pYES2R  *MoARF1*qRTF  *MoARF1*qRTR  *MoARF6*qRTF  *MoARF6*qRTR  *MoARL1*qRTF  *MoARL1*qRTR  *MoARL3*qRTF  *MoARL3*qRTR  *MoARL8*qRTF  *MoARL8*qRTR  *MoCIN4*qRTF  *MoCIN4*qRTR  *MoSAR1*qRTF  *MoSAR1*qRTR  *ACTIN*F  *ACTIN*R  *MoARF1*UF  *MoARF1*UR  *MoARF1*DF  *MoARF1*DR  *MoARF6*UF  *MoARF6*UR  *MoARF6*DF  *MoARF6*DR  *MoARL1*UF  *MoARL1*UR  *MoARL1*DF  *MoARL1*DR  *MoARL3*UF  *MoARL3*UR  *MoARL3*DF  *MoARL3*DR  *MoARL8UF*  *MoARL8UR*  *MoARL8DF*  *MoARL8DR*  *MoCIN4*UF  *MoCIN4UR*  *MoCIN4DF*  *MoCIN4DR*  *MoSAR1*UF  *MoSAR1*UR  *MoSAR1*DF  *MoSAR1*DR  *MoARF1*CPRUF  *MoARF1*CPRUR  *MoARF1*CPRDF  *MoARF1*CPRDR  *MoSAR1*CPRUF  *MoSAR1*CPRUR  *MoSAR1*CPRDF  *MoSAR1*CPRDR  P*MoNIA1*F  P*MoNIA1*R  *MoARF1*CPRNBF  *MoARF1*CPRNBR  *MoARF1*CPRBWF  *MoSAR1*CPRNBF  *MoSAR1*CPRNBR  *MoSAR1*CPRBWF  MoArl1^Q71LΔ17N^ADF1  MoArl1^Q71LΔ17N^ADR1  MoArl1^Q71LΔ17N^ADF2  MoArl1^Q71LΔ17N^ADR2  MoArl1^T31NΔ17N^ADF1  MoArl1^T31NΔ17N^ADR1  MoArl1^T31NΔ17N^ADF2  MoArl1^T31NΔ17N^ADR2  MoArf1^Q71LΔ17N^ADF1  MoArf1^Q71LΔ17N^ADR1  MoArf1^Q71LΔ17N^ADF2  MoArf1^Q71LΔ17N^ADR2  MoArf1^T31NΔ17N^ADF1  MoArf1^T31NΔ17N^ADR1  MoArf1^T31NΔ17N^ADF2  MoArf1^T31NΔ17N^ADR2  MoCin4^Q68LΔ14N^ADF1  MoCin4^Q68LΔ14N^ADR1  MoCin4^Q68LΔ14N^ADF2  MoCin4^Q68LΔ14N^ADR2  MoArl3^Q82LΔ17N^ADF1  MoArl3^Q82LΔ17N^ADR1  MoArl3^Q82LΔ17N^ADF2  MoArl3^Q82LΔ17N^ADR2  MoArl8^Q75LΔ20N^ADF1  MoArl8^Q75LΔ20N^ADR1  MoArl8^Q75LΔ20N^ADF2  MoArl8^Q75LΔ20N^ADR2  MoArf6^Q71LΔ17N^ADF1  MoArf6^Q71LΔ17N^ADR1  MoArf6^Q71LΔ17N^ADF2  MoArf6^Q71LΔ17N^ADR2  MoSar1^H74GΔ20N^ADF1  MoSar1^H74GΔ20N^ADR1  MoSar1^H74GΔ20N^ADF2  MoSar1^H74GΔ20N^ADR2  MoArf1NBF:  MoArf1NBR:  MoArf1BWF:  MoArf6NBF:  MoArf6NBR:  MoArf6BWF:  MoArl1NBF:  MoArl1NBR:  MoArl1BWF:  MoArl3NBF:  MoArl3NBR:  MoArl3BWF:  MoArl8NBF:  MoArl8NBR:  MoArl8BWF:  MoCin4NBF:  MoCin4NBR:  MoCin4BWF:  MoSar1NBF:  MoSar1NBR:  MoSar1BWF:  HPHR | | GCTCTAGATCACTTCTTGGCCGGCGCAG  CGGGATCCATGGGACAAAGTGTTTCATGGCTTT  GCTCTAGATTATGACTCTTGCGAGACCGTTTGC  CGGAATTCATGTATCACCTAGCTAAAGGAC  GCTCTAGATCACCTCATTACAGGCGGTCT  CGGGATCCATGTTGTCAATCCTCAGGAAAGC  GCTCTAGACTAATATAGGAACAACCTAGC  TGGGGGAAGAAGGAGATGAGAA  GAAGCCGATGGTAGGAATGGTG  GATTCGTCCTCTGTGGAGGCATTA  GCTGTCCTTCATCTCTCGGTCATT  TGACCGTTGCCCTGTCTTCCTC  GTCTGACCACCCAAGTCCCATA  CCAGCAAGGAAGAGTATTCGGTGATAT  TCGCCGCCAGGGTTGAAGAGGT  CGAATTTACCATAGACACGATCCCCACT  GCCACGGCAATACCTCTCCCAC  CAGGAAAGCACGGCTGAAAGAC  CTAGGGTGGGGCTGACGGTATT  GGGCAACAAGATCGACCACCCC  CATAACTACGGAGCACATGAACACCTC  CCATGTACCCTGGTCTTTCG  TTCGAGATCCACATCTGCTG  GGGGTACCCAATGGGTGGTTGAAAAATGATG  CCGCTCGAGGCGACAGGAACAAAAACAGGAC  GGACTAGTCCAAGTTTTCCATCAGAACAATCG  GCGAGCTCAATACCAACTAAACACCGTCTCCGT  ACGCGTCGACTTGTTCTATTGAAGCGGTAAAGTG  CGGAATTCGGAAGCCTCGTGTGTATGTGTT  CGGGATCCTGGGATTATGACCGCCTTCTTT  GGACTAGTCAGCGCCAACTGCTTCGAG  GGGGTACCCCCTCATCCCACCATCCTG  CCCAAGCTTGAAGATACGGAGCTTTGCGGT  CGGGATCCTGGTATTACACTTCACCCACACG  GGACTAGTGCCTGGTTCAGACTGCTTGC  ACGCGTCGACCGTGACGACCTCGACGACATACT  CGGAATTCGATACGGTGCCGCATTGACTT  CGGGATCCGCTGGTGCCTATTGTGTTACGAT  GGACTAGTGACTTGACGGGCGTGATATAGAC  ACGCGTCGACCTTGGGGACCGTGTCGCTACT  CGGAATTCGGGGGTGTCTATTCTCACTTGAACT  GGACTAGTTAGTAATGGCTCCAGTTGGTGACAG  GCGAGCTCTGACTTTTGTAGCCCACATTCCG  CCGCTCGAGGGAGACGGGCATCAAAACCTAA  CGGAATTCCTTGCCTGCTGAAGGGATGGAAT  CGGGATCCAACTAATGACCCAGCATGTGGACGG  GGACTAGTAGTGTTTGTCGGGCGAGAGTGG  ACGCGTCGACGTAGCAACCTGATGGCCCGAGAT  CGGAATTCTGTGACAATCGTTTGTTCCGCCGCG  CGGGATCCAGGAAGAAAGATACCAAGTTGCAAG  GGACTAGTTCGGCGGTCTGATGTTGTC  CCGCTCGAGCCAAACTCCCTGTCGCCAACCT  CGGAATTCTATCATCACGTAAGTGCGGTGAGCA  CTGGTTTCCTACACATATTCCAACCATGGGTCTCAGCATCAGCAAGCTGT  GGACTAGTTGGGGTGTTGTTGAAGAGGAAGGA  CCGCTCGAGCTTGGGCGTCTGTTGGTATTTT  CGGAATTCAACTATCCTTAACATCTTGGAATCC  CTGGTTTCCTACACATATTCCAACCATGTGGATCATCAACTGGTTTTACG  GGACTAGTCGGATTAAACAGCCGTACCATC  CGGGATCCATCTTTGGTCGTCCAATCCCCTGGG  GGTTGGAATATGTGTAGGAAACCAG  ACCTTGCCTCGCCCAGTAGTAGTTT  GGGGTCAGTTGGCCGTTTGATT  GGTGCCCCATACCCAAAAGTCA  CGTTCCAACTATATGGGGTTTT  CTTCTTACTGGGTACTGATTAGGG  AACGGGTTTCACTGGGCTGCTA  CCGGAATTCATGATCAGGATTCTGATCCTCGGCCTG  CAGACCACCCAAGTCCCATACGTTG  AACGTATGGGACTTGGGTGGTCTGACATCGATCCGTCCCTACTGGCGAT  CGCGGATCCTTATGACTCTTGCGAGACCGTTTG  CCGGAATTCATGATCAGGATTCTGATCCTCGGCCTG  GTTTTTGCCTGCATTGTCCTGACC  GTCAGGACAATGCAGGCAAAAACACATTGCTGTATAGGTTGAAGGTAT  CGCGGATCCTTATGACTCTTGCGAGACCGTTTG  CGGAATTCATGATGAGAATTTTGATGGTCGGTCTCG  GCCACCCACATCCCACAC  GCCACCCACATCCCACACCTGGACAAGATTCGTCCTCTGTGGA  CGGGATCCTTACTGGTGGCCTGCCTTGC  CGGAATTCATGAGAATTTTGATGGTC  GTTTTTACCGGCGGCAT  ATGCCGCCGGTAAAAACACGATTCTGTACAAGC  CGGGATCCTTACTGGTGGCCTGCCTTGC  CGGAATTCATGCTACGGATTTTGATGCTTGGGCTGG  GCCGCCGACGTCCCATATGTTCAAC  CATATGGGACGTCGGCGGCCTAAAGACATTGAGATCTTACTGGA  CGGGATCCCTAATATAGGAACAACCTAGCTTTT  CGCCATATGATGTATTCGGTGATATTACTCGGGC  CCCGCCCACGTCCCAGATCTTCATA  ATCTGGGACGTGGGCGGGCTGCACAGCCTGCGTAAGCTGT  CCGGAATTCTCACCTCATTACAGGCGGTCT  CGCCATATGATGATGGACGTGACCATGATTGGAC  TCCCCCCATATCCCAGCATTTCATT  TGCTGGGATATGGGGGGACTGGAACGGTTCCGCGGCAT  CCGGAATTCTCATTTGCTAGAGTACTTCATCAAC  CGCCATATGATGATGCGGCTGCTGATGCT  ACCACCGACATCCCAGACGTT  GTCTGGGATGTCGGTGGTCTGGACAAGATTCGTCCTCTGTGGA  CCGGAATTCTCACTTCTTGGCCGGCGCAG  TACGACGTACCAGATTACGCTCATATGATGGCGAAGCTGCTTTTCCTCGGTC  ACCGCCCAGGTCAAAGGTGGTAAAG  CCACCTTTGACCTGGGCGGTGGTCAGCAGGCCCGCCGCCT  TCGATGCCCACCCGGGTGGAATTCCTACACATACTGCGAAAGCCATCTG  GCTGTGGGGGAAGAAGGAGAT  TCAAAATACGAGCGGACCAGG  GACTGGTGGATTGTCAAGGGTGT  AAGGAGATGCGGCTGCTGAT  GCAGACCCTCTCCCGTGG  CAAGCATTTGGTGAACTACCCGC  GACCGTTGCCCTGTCTTCCT  CGTCCAACCCAAGGCAGAGT  CTTGGACTCTGTGGGCTGTACTT  TTCGGTGATATTACTCGGGCTCG  CCTCATTACAGGCGGTCTGCTCT  TACAACAACCTGCTCACCATCCC  TGCCTGCCTCATCTTTCCTGG  CTTCATCAACCATTGGATAACCGC  TAGAGCCTCCTTTGTTTGAGTGGGT  CGTCAATACCGTCAGCCCCACC  TGAGATTCTTCCCCGTCATAGC  GGTCTGTGGTGAGGTTGAATAGGGT  CCATCCTCCAGCCTACTCTCCA  TTAAGCCGCCATAGGTAAAATGC  CGTGAGTTTTGCTGGGCCT  CGCTACTGCTACAAGTGGGGCT | amplify *MoARF6* sequence  amplify *MoARL1* sequence  amplify *MoARL1* sequence  amplify *MoARL3*sequence  amplify *MoARL3* sequence  amplify *MoCIN4* sequence  amplify *MoCIN4* sequence  QRT-PCR Primer of *MoARF1*  QRT-PCR Primer of *MoARF1*  QRT-PCR Primer of *MoARF6*  QRT-PCR Primer of *MoARF6*  QRT-PCR Primer of *MoARL1*  QRT-PCR Primer of *MoARL1*  QRT-PCR Primer of *MoARL3*  QRT-PCR Primer of *MoARL3*  QRT-PCR Primer of *MoARL8*  QRT-PCR Primer of *MoARL8*  QRT-PCR Primer of *MoCIN4*  QRT-PCR Primer of *MoCIN4*  QRT-PCR Primer of *MoSAR1*  QRT-PCR Primer of *MoSAR1*  QRT-PCR Primer of *ACTIN*  QRT-PCR Primer of *ACTIN*  amplify *MoARF1* 5’ flank sequence  amplify *MoARF1* 5’ flank sequence  amplify *MoARF1* 3’ flank sequence  amplify *MoARF1* 3’ flank sequence  amplify *MoARF6* 5’ flank sequence  amplify *MoARF6* 5’ flank sequence  amplify *MoARF6* 3’ flank sequence  amplify *MoARF6* 3’ flank sequence  amplify *MoARL1* 5’ flank sequence  amplify *MoARL1* 5’ flank sequence  amplify *MoARL1* 3’ flank sequence  amplify *MoARL1* 3’ flank sequence  amplify *MoARL3* 5’ flank sequence  amplify *MoARL3* 5’ flank sequence  amplify *MoARL3* 3’ flank sequence  amplify *MoARL3* 3’ flank sequence  amplify *MoARL8* 5’ flank sequence  amplify *MoARL8* 5’ flank sequence  amplify *MoARL8* 3’ flank sequence  amplify *MoARL8* 3’ flank sequence  amplify *MoCIN4* 5’ flank sequence  amplify *MoCIN4* 5’ flank sequence  amplify *MoCIN4* 3’ flank sequence  amplify *MoCIN4* 3’ flank sequence  amplify *MoSAR1* 5’ flank sequence  amplify *MoSAR1* 5’ flank sequence  amplify *MoSAR1* 3’ flank sequence  amplify *MoSAR1* 3’ flank sequence  amplify *MoARF1*(CPR) 5’ flank sequence  amplify *MoARF1*(CPR) 5’ flank sequence  amplify *MoARF1*(CPR) 3’ flank sequence  amplify *MoARF1*(CPR) 3’ flank sequence  amplify *MoARF1*(CPR) 5’ flank sequence  amplify *MoARF1*(CPR) 5’ flank sequence  amplify *MoARF1*(CPR) 3’ flank sequence  amplify *MoARF1*(CPR) 3’ flank sequence  amplify *MoNIA1* promoter  amplify *MoNIA1* promoter  amplify *MoARF1(*CPR*)* probe sequence  amplify *MoARF1(*CPR*)* probe sequence  validation of *MoARF1(*CPR*)* deletion  amplify *MoSAR1(*CPR*)* probe sequence  amplify *MoSAR1*CPR*)* probe sequence  validation of *MoSAR1(*CPR*)* deletion  Construct pGADT7-MoArl1^Q71LΔ17N^  Construct pGADT7-MoArl1^Q71LΔ17N^  Construct pGADT7-MoArl1^Q71LΔ17N^  Construct pGADT7-MoArl1^Q71LΔ17N^  Construct pGADT7-MoArl1^T31NΔ17N^  Construct pGADT7-MoArl1^T31NΔ17N^  Construct pGADT7-MoArl1^T31NΔ17N^  Construct pGADT7-MoArl1^T31NΔ17N^  Construct pGADT7-MoArf1^Q71LΔ17N^  Construct pGADT7-MoArf1^Q71LΔ17N^  Construct pGADT7-MoArf1^Q71LΔ17N^  Construct pGADT7-MoArf1^Q71LΔ17N^  Construct pGADT7-MoArf1^T31NΔ17N^  Construct pGADT7-MoArf1^T31NΔ17N^  Construct pGADT7-MoArf1^T31NΔ17N^  Construct pGADT7-MoArf1^T31NΔ17N^  Construct pGADT7-MoCin4^Q68LΔ14N^  Construct pGADT7-MoCin4^Q68LΔ14N^  Construct pGADT7-MoCin4^Q68LΔ14N^  Construct pGADT7-MoCin4^Q68LΔ14N^  Construct pGADT7-MoArl3^Q82LΔ17N^  Construct pGADT7-MoArl3^Q82LΔ17N^  Construct pGADT7-MoArl3^Q82LΔ17N^  Construct pGADT7-MoArl3^Q82LΔ17N^  Construct pGADT7-MoArl8^Q75LΔ20N^  Construct pGADT7-MoArl8^Q75LΔ20N^  Construct pGADT7-MoArl8^Q75LΔ20N^  Construct pGADT7-MoArl8^Q75LΔ20N^  Construct pGADT7-MoArf6^Q71LΔ17N^  Construct pGADT7-MoArf6^Q71LΔ17N^  Construct pGADT7-MoArf6^Q71LΔ17N^  Construct pGADT7-MoArf6^Q71LΔ17N^  Construct pGADT7-MoSar1^H74GΔ20N^  Construct pGADT7-MoSar1^H74GΔ20N^  Construct pGADT7-MoSar1^H74GΔ20N^  Construct pGADT7-MoSar1^H74GΔ20N^  amplify *MoARF1* probe sequence  amplify *MoARF1* probe sequence  validation of *MoARF1* deletion  amplify *MoARF6* probe sequence  amplify *MoARF6* probe sequence  validation of *MoARF6* deletion  amplify *MoARL1* probe sequence  amplify *MoARL1* probe sequence  validation of *MoARL1* deletion  amplify *MoARL3* probe sequence  amplify *MoARL3*probe sequence  validation of *MoARL3* deletion  amplify *MoARL8* probe sequence  amplify *MoARL8* probe sequence  validation of *MoARL8* deletion  amplify *MoCIN4* probe sequence  amplify *MoCIN4* probe sequence  validation of *MoCIN4* deletion  amplify *MoSAR1* probe sequence  amplify *MoSAR1* probe sequence  validation of *MoSAR1* deletion  validation of Gene deletion |
| FL1111 | GGAGGTCAACACATCAATG | | amplify HPH sequence |
| FL1112  pYF11*MoARF1*F  pYF11*MoARF1*R  pYF11*MoARF6*F  pYF11*MoARF6*R  pYF11*MoARL1*F  pYF11*MoARL1*R  pYF11*MoARL3*F  pYF11*MoARL3*R  pYF11*MoCIN4*F  pYF11*MoCIN4*R  pRP27*MoCIN4*F  pYF11*MoSAR1*F  pYF11*MoSAR1*R  pYF11*MoARL8*F  pYF11*MoARL8*R  p*MoCIN4^T28N^*R1  p*MoCIN4^T28N^*F2  p*MoCIN4^Q68L^*R1  p*MoCIN4^Q68L^*F2  p*MoCIN4^N123I^*R1  p*MoCIN4^N123I^*F2  p*MoARL1^T31N^*R1  p*MoARL1^T31N^*F2  p*MoARL1^Q71L^*R1  p*MoARL1^Q71L^*F2  p*MoARL1^N126I^*R1  p*MoARL1^N126I^*F2  p*MoARL1^G2A^*R1  p*MoARL1^G2A^*F2  pHZ68-*MoARF1*F  PHZ68-*MoARF1*R  pHZ68-*MoARL1*F  pHZ68-*MoARL1*R  pXY203-*MoARF1*F  pXY203-*MoARF1*R  pXY203-*MoARF6*F  pXY203-*MoARF6*R  pXY203-*MoARL1*F  pXY203-*MoARL1*R  pXY203-*MoARL3*F  pXY203-*MoARL3*R  pXY203-*MoGGA1*F  pXY203-*MoGGA1*R  pXY203-*MoSAR1*F  pXY203-*MoSAR1*R  pXY203-*MoARL8*F  pXY203-*MoARL8*R  pXY203-*MoCIN4*F  pXY203-*MoCIN4*R  *MoSFT2*-RFPF1  *MoSFT2*-RFPR1  *MoSFT2*-RFPF2  *MoSFT2*-RFPR2  pHZ65-*MoGGA1*F  pHZ65-*MoGGA1*R  pYF11*MoGGA1*F  pYF11*MoGGA1*R  *MoGGA1*UF  *MoGGA1*UR  *MoGGA1*DF  *MoGGA1*DR  *MoGGA1*NBF  *MoGGA1*NBR  *MoGGA1*BWF | CTCTATTCCTTTGCCCTCG  ACTCACTATAGGGCGAATTGGGTACTCAAATTGGTTTGGGTGGTTGAAAAATGATGAA  CACCACCCCGGTGAACAGCTCCTCGCCCTTGCTCACCTGGTGGCCTGCCTTGCG  ACTCACTATAGGGCGAATTGGGTACTCAAATTGGTTGCGTGGTCGGCGAGACGTGTCTG  CACCACCCCGGTGAACAGCTCCTCGCCCTTGCTCACCTTCTTGGCCGGCGCAGGCG  ACTCACTATAGGGCGAATTGGGTACTCAAATTGGTTAGGTCCCGGTCCTGGGCTTTTAG CACCACCCCGGTGAACAGCTCCTCGCCCTTGCTCACTGACTCTTGCGAGACCGTTTGCT  ACTCACTATAGGGCGAATTGGGTACTCAAATTGGTTGAGGATTGGTCAGCTGCTAACTTGG  CACCACCCCGGTGAACAGCTCCTCGCCCTTGCTCACCCTCATTACAGGCGGTCTGCTCTC  ACTCACTATAGGGCGAATTGGGTACTCAAATTGGTTCAAGTGATTGTAAGGGAAGAAAC  CACCACCCCGGTGAACAGCTCCTCGCCCTTGCTCACATATAGGAACAACCTAGCTTTTG  TTTCGTAGGAACCCAATCTTCAAAATGTTGTCAATCCTCAGGAAAGCAC  ACTCACTATAGGGCGAATTGGGTACTCAAATTGGTTAACAGACAACCGACAGACAGCC  CACCACCCCGGTGAACAGCTCCTCGCCCTTGCTCACCACATACTGCGAAAGCCATCTGATA  ACTCACTATAGGGCGAATTGGGTACTCAAATTGGTTACCCTCGTTGGCTCCCTCCG  CACCACCCCGGTGAACAGCTCCTCGCCCTTGCTCACTTTGCTAGAGTACTTCATCAACCATTGG  CTTGCCAGCATTGTCCAGCCCACTT  AAGTGGGCTGGACAATGCTGGCAAG AACACCATCGTCAAAAAAATCATGG  GCCGCCGACGTCCCCTGGAAT  ATTCCAGGGGACGTCGGCGGC CTAAAGACATTGAGATCTTACTGGA  GGCAAAGACCAGAAGACTGGCT  AGCCAGTCTTCTGGTCTTTGCC ATCAAAACCGATGTAAATGGTTGC  GTTTTTGCCTGCATTGTCCTGACC  GTCAGGACAATGCAGGCAAAAACACATTGCTGTATAGGTTGAAGGTAT  CAGACCACCCAAGTCCCATACGTTG  AACGTATGGGACTTGGGTGGTCTGACATCGATCCGTCCCTACTGGCGAT  GATTGCAAAGACCAGCAATGCT  GGATGCAGCATTGCTGGTCTTTGCAATCAAGCAGGATCAGCCTGGAGCAAAG  CATTGTGCTCGTTTGTGTGTTGCCC  GGCAACACACAAACGAGCACAATGGCACAAAGTGTTTCATGGCTTTCAG  ACTCACTATAGGGCGAATTGGGTACTCAAATTGGTTTGGGTGGTTGAAAAATGATGAA  GTTCGGGATCTTGCAGGCCGGGCGCTGGTGGCCTGCCTTGCG  ACTCACTATAGGGCGAATTGGGTACTCAAATTGGTTATTTCGAGTTCCATTTTGCCCATA  GTTCGGGATCTTGCAGGCCGGGCGTGACTCTTGCGAGACCGTTTGCTAA  ACTCACTATAGGGCGAATTGGGTACTCAAATTGGTTTGGGTGGTTGAAAAATGATGAA  TTCGAATTTAGCAGCAGCGGTTTCTTTCTGGTGGCCTGCCTTGCG  ACTCACTATAGGGCGAATTGGGTACTCAAATTGGTTGCGTGGTCGGCGAGACGTGTCTG  ACTCACTATAGGGCGAATTGGGTACTCAAATTGGTTGCGTGGTCGGCGAGACGTGTCTG  ACTCACTATAGGGCGAATTGGGTACTCAAATTGGTTAGGTCCCGGTCCTGGGCTTTTAG  TTCGAATTTAGCAGCAGCGGTTTCTTTTGACTCTTGCGAGACCGTTTGCTAA  ACTCACTATAGGGCGAATTGGGTACTCAAATTGGTTGAGGATTGGTCAGCTGCTAACTTGG  TTCGAATTTAGCAGCAGCGGTTTCTTTCCTCATTACAGGCGGTCTGCTCTC  ACTCACTATAGGGCGAATTGGGTACTCAAATTGGTTGGCGACAAGGTAGGGCAGCAGG  TTCGAATTTAGCAGCAGCGGTTTCTTTTGCCACCTTGATCTCCGAAACCT  ACTCACTATAGGGCGAATTGGGTACTCAAATTGGTTAACAGACAACCGACAGACAGCC  TTCGAATTTAGCAGCAGCGGTTTCTTTCACATACTGCGAAAGCCATCTGATA  ACTCACTATAGGGCGAATTGGGTACTCAAATTGGTTACCCTCGTTGGCTCCCTCCG  TTCGAATTTAGCAGCAGCGGTTTCTTTTTTGCTAGAGTACTTCATCAACCATTGG  TTTCGTAGGAACCCAATCTTCAAAATGTTGTCAATCCTCAGGAAAGCAC  TTCGAATTTAGCAGCAGCGGTTTCTTTATATAGGAACAACCTAGCTTTTG  ACTCACTATAGGGCGAATTGGGTACTCAAATTGGTTATTTTGAGGGTGAACAAACATCC  GCCAGTCATCCAAGCGGC  GCCGCTTGGATGACTGGCATGGCCTCCTCCGAGGACGTCATCA  CACCACCCCGGTGAACAGCTCCTCGCCCTTGCTCACTTAGGCGCCGGTGGAGTGGCGGC  ACTCACTATAGGGCGAATTGGGTACTCAAATTGGTTGGCGACAAGGTAGGGCAGCAGG  GCTCACCATCGTGGCGATGGAGCGTGCCACCTTGATCTCCGAAACCT  ACTCACTATAGGGCGAATTGGGTACTCAAATTGGTTGGCGACAAGGTAGGGCAGCAGG  CACCACCCCGGTGAACAGCTCCTCGCCCTTGCTCACTGCCACCTTGATCTCCGAAACCT  CCGCTCGAGCGGCTGTTGCTGCTGCTGTCGTT  CCGGAATTCTGTGAAAGGGTGTTTGGGCGTGAGG  CGCGGATCCCCTCGCCTCACGGCAAGCCTTCATA  GGACTAGTAGCCTCTCGAATGGCATCCTCTT  CCCGGCTTTACTGTCGTCCATA  CACACTTCGAGAACCTGCGTGA  CACCGTAGGAAAATGGCTCGAGGAT | | amplify HPH sequence  Construction of *MoARF1-*GFP  Construction of *MoARF1-*GFP  *MoARF6* complementation  *MoARF6* complementation  *MoARL1* complementation  *MoARL1* complementation  *MoARL3* complementation  *MoARL3* complementation  *MoCIN4* complementation  *MoCIN4* complementation  Construct RP27-*MoCIN4*  *MoCIN4* complementation  *MoCIN4* complementation  Construct pYF11-*MoARL8*  Construct pYF11-*MoARL8*  Construct pYF11-*CIN4^T28N^*  Construct pYF11-*CIN4^T28N^*  Construct pYF11-*CIN4^Q68L^*  Construct pYF11-*CIN4^Q68L^*  Construct pYF11-*CIN4^N123I^*  Construct pYF11-*CIN4^N123I^*  Construct pYF11-*ARL1^T31N^*  Construct pYF11-*ARL1^T31N^*  Construct pYF11-*ARL1^Q71L^*  Construct pYF11-*ARL1^Q71L^*  Construct pYF11-*ARL1^N126I^*  Construct pYF11-*ARL1^N126I^*  Construct pYF11-*ARL1^G2A^*  Construct pYF11-*ARL1^G2A^*  Construction of *MoARF1-*YFP^C^  Construction of *MoARF1-*YFP^C^  Construction of *MoARL1-*YFP^C^  Construction of *MoARL1-*YFP^C^  Construction of *MoARF1-*S  Construction of *MoARF1-*S  Construction of *MoARF6-*S  Construction of *MoARF6-*S  Construction of *MoARL1-*S  Construction of *MoARL1-*S  Construction of *MoARL3-*S  Construction of *MoARL3-*S  Construction of *MoGGA1-*S  Construction of *MoGGA1-*S  Construction of *MoSAR1-*S  Construction of *MoSAR1-*S  Construction of *MoARL8-*S  Construction of *MoARL8-*S  Construction of *MoCIN4-*S  Construction of *MoCIN4-*S  Construction of *MoSFT2-*RFP  Construction of *MoSFT2-*RFP  Construction of *MoSFT2-*RFP  Construction of *MoSFT2-*RFP  Construction of *MoGGA1-*YFP^N^  Construction of *MoGGA1-*YFP^N^  *MoGGA1* complementation  *MoGGA1* complementation  amplify *MoGGA1* 5’ flank sequence  amplify *MoGGA1* 5’ flank sequence  amplify *MoGGA1* 3’ flank sequence  amplify *MoGGA1* 3’ flank sequence  amplify *MoGGA1* probe sequence  amplify *MoGGA1* probe sequence  validation of *MoGGA1* deletion |
| MoGga1BDF | CGGAATTCATGGAGGCGGCATCCGCCCGAT | | Construct pGBKT7-MoGga1 |
| MoGga1BDR | CGGGATCCTCATGCCACCTTGATCTCCGAAACC | | Construct pGBKT7-MoGga1 |
| MoGga1^I208N^BDR1 | AAGCTCCTGTAGTTTTGCCGATTGC | | Construct pGBKT7-MoGga1^I208N^ |
| MoGga1^I208N^BDF2 | CGGCAAAACTACAGGAGCTTAACCGGCGAGGAACCCCAGAGGACT | | Construct pGBKT7-MoGga1^I208N^ |
| MoGga1^GAT^BDF | CGGAATTCGATGATGCTGCAGTGCTGAATCCTA | | Construct pGBKT7-MoGga1^GAT^ |
| MoGga1^GAT^BDR | CGGGATCCCGACGCCCCGGCAGCGG | | Construct pGBKT7-MoGga1^GAT^ |
| p*MoGGA1*^I208N^R1 | AAGCTCCTGTAGTTTTGCCGATTGC | | Construct pYF11-*MoGGA1*^I208N^ |
| p*MoGGA1*^I208N^F2 | CGGCAAAACTACAGGAGCTTAACCGGCGAGGAACCCCAGAGGACT | | Construct pYF11-*MoGGA1*^I I208N^ |
| p*MoGGA1*^GAT^R1 | CATTGTGAAAGGGTGTTTGGGCGTG | | Construct pYF11-*MoGGA1*^GAT^ |
| p*MoGGA1*^GAT^F2 | CCAAACACCCTTTCACAATGGATGATGCTGCAGTGCTGAATCCTA | | Construct pYF11-*MoGGA1*^GAT^ |
